# Proposed Method for Mass Spectrometric Measurement of the Gonadotropin-Releasing Hormone Agonist Deslorelin

**DOI:** 10.3390/toxics14100913

**Published:** 2026-10-16

**Authors:** Christine Wei, John P. Buchweitz, Rinosh Mani, Dalen Agnew, Andreas F. Lehner

**Affiliations:** 1 Department of Comparative Medicine and Integrative Biology, College of Veterinary Medicine, Michigan State University, East Lansing, MI 48824, USA; weizimu@msu.edu; 2 Pathobiology & Diagnostic Investigation, College of Veterinary Medicine, Michigan State University, East Lansing, MI 48824, USA; buchwei2@msu.edu (J.P.B.); agnewd@msu.edu (D.A.); 3 Michigan State University Veterinary Diagnostic Laboratory, Section of Toxicology, College of Veterinary Medicine, Michigan State University, 4125 Beaumont Rd, East Lansing, MI 48910, USA; 4 Michigan State University Veterinary Diagnostic Laboratory, Section of Bacteriology, Michigan State University, East Lansing, MI 48910, USA; manirino@msu.edu

**Keywords:** deslorelin, enzymatic cleavage, Clostridiopeptidase B, gas chromatography tandem mass spectrometry, matrix-assisted laser desorption/ionization–time-of-flight mass spectrometry, gonadotropin-releasing hormone

## Abstract

Deslorelin (C_64_H_83_N_17_O_12_) is a peptide GnRH agonist drug with monoisotopic mass 1281.64 Da applied as a drug-releasing implant in veterinary medicine for promoting ovulation and male/female contraception. Despite relative safety, it is suspected of reproductive toxicity and potential harm to unborn or breast-fed children. The compound was designed for high potency and prolonged half-life making it difficult to measure quantitatively in serum. A deslorelin LC-MS/MS method relies on detecting unmetabolized undegraded intact peptide, a crucial consideration since certain metabolites are thought to have activity. We propose a method for analyzing deslorelin indirectly by cleaving a unique portion of the carboxy terminus containing the modified proline ProNHEt (N-ethyl-2-pyrrolidine carboxamide), which can be removed by Clostridiopeptidase specific for arginine bonds. ProNHEt can be extracted from digested serum, reacted to an N-t-butyldimethylsilyl derivative and analyzed by GC-MS/MS. Settings were optimized for detectability to <1 ppb, and derivatization gave maximum yields by adjustment of solvent, reaction time, temperature and additives. MALDI-TOF applied to deslorelin revealed binding by copper. The proposed method can be used to measure deslorelin in serum samples from various zoo and small animals. Detection of deslorelin by examination of released ProNHEt is proposed to have applicability to other GnRH superagonists such as triptorelin and deslorelin breakdown products that exhibit the ProNHEt structure.

## 1. Introduction

**Figure 1:**
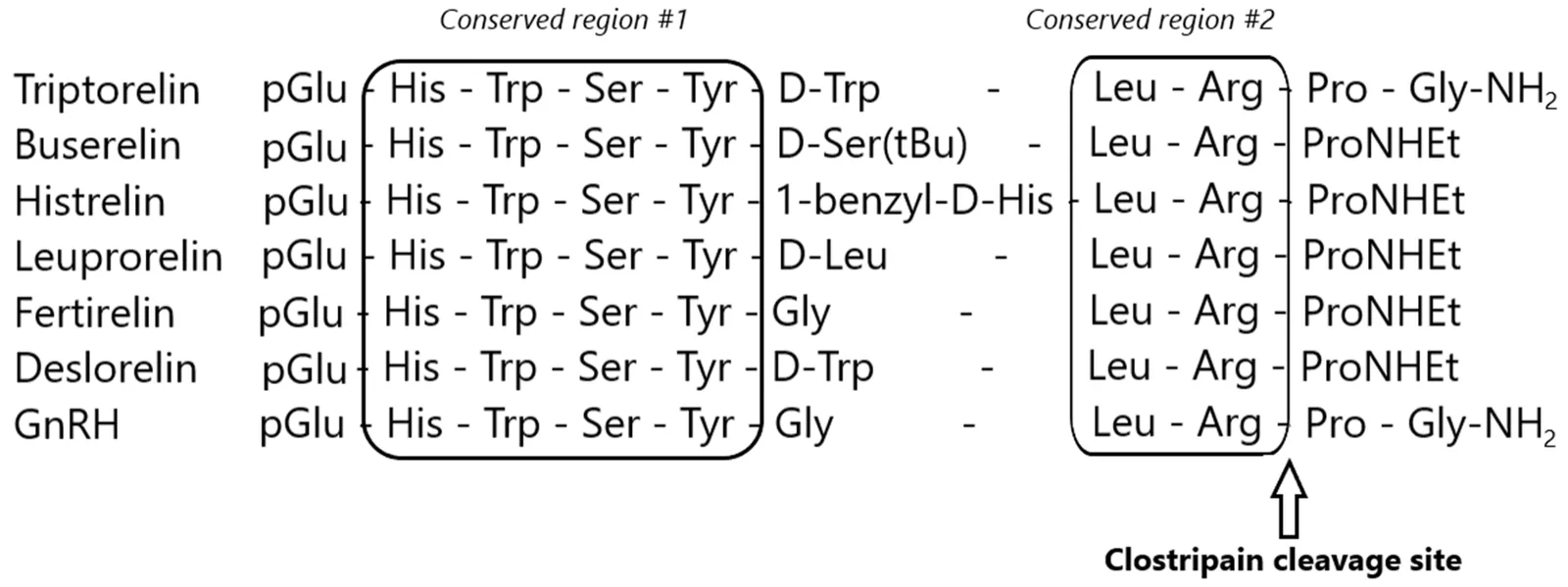
Peptide sequence comparisons of gonadotropin analogs Triptorelin, Buserelin, Histrelin, Leuprorelin, Fertirelin and Deslorelin with natural GnRH. The arrow indicates the site of cleavage by clostripain. Buserelin, Histrelin, Leuprorelin, Fertirelin and Deslorelin would release ProNHEt on treatment with clostripain, whereas Triptorelin or GnRH would eject a ProNH-acetamido group not recognized by the described method. Sequences are from PubChem (https://pubchem.ncbi.nlm.nih.gov/ (Accessed 28 August 2026)). Amino acids are L-configuration unless otherwise labeled. Unusual amino acids include pGlu = pyroglutamic acid; D-Ser(tBu) = t-butyl derivative of D-Ser; ProNHEt = N-ethyl-2-pyrrolidinecarboxamide; 1-benzyl-D-His = 1-benzyl derivative of D-His.

Deslorelin is a peptide with molecular formula C_64_H_83_N_17_O_12_ (monoisotopic mass 1281.64 Da), which is available as an injectable or implantable synthetic gonadotropin-releasing hormone (GnRH) agonist active at pituitary GnRH receptors [1]. The amino acid sequence of deslorelin is H-Pyr-His-Trp-Ser-Tyr-Trp-Leu-Arg-Pro-NHEt, which includes three unusual amino acids H-Pyr (L-Pyroglutamic Acid), D-trp and ProNHEt (N-ethyl-2-pyrrolidinecarboxamide). GnRH itself ordinarily originates in the hypothalamic region and regulates gonadotropin follicle-stimulating hormone (FSH) and luteinizing hormone (LH) secreted from the anterior pituitary gland thereby influencing reproductive functions. GnRH expresses its effects in peripheral tissues particularly gonads (ovaries and testes) [2]. As an analog of GnRH, deslorelin has engineered substitutions in its amino acid sequence, and it has a significant role in terms of the hormonal regulation of reproduction by influencing FSH and LH release from the pituitary [3]. Deslorelin structure is shown in Figure 1 in comparison to other available GnRH analogs. It is currently approved for use in veterinary medicine as a contraceptive. Its high potency makes it prone to over- or underdosing, making therapeutic drug monitoring essential to avoid toxicity while maintaining activity.

Deslorelin can be administered subcutaneously as an implant in a controlled-release formulation, or it can be administered as a bioresorbable pellet. It is primarily used to control behavioral problems associated with reproductive issues, but additional therapeutic applications include treatment of endometriosis, uterine leiomyoma, and malignant neoplasms. Clinically, deslorelin implants are used as contraception in male cats and induce fertile estrus in bitches [4], while deslorelin acetate is being studied as a means of estrus suppression in the mare [5]. However, evidence has accumulated suggesting that the duration of deslorelin effect is much longer than expected [5,6,7,8,9]. In a study by Herbert et al. [10], deslorelin implants displayed capacity for control of reproduction in wallabies. In cats, contraceptive effects lasted much longer than 6 to 12 months, resulting in delayed recovery of reproductive function [11]. Ferre’-Dolcet et al. [12] found that the resumption of ovarian function in feline queens needed 3–7 weeks post-removal of a 4.7 mg deslorelin implant. The recovery of reproductive function in various species continues to be investigated. In the domestic cat, cumulus-oocyte complex recovery and embryo production are negatively affected by deslorelin implantation in the subcutaneous tissue [11]. Similarly, an observation by scientists at the Saint Louis Zoo (Missouri, USA) determined that the infertility of zoo animals persisted for up to ten years following deslorelin implantation [13]. It is therefore considered of value to quantitate low-level deslorelin incurred by long-term implants.

Current quantitative approaches to deslorelin in serum include enzyme-linked immunosorbent assay (ELISA). The ELISA principle in brief: the antiserum raised against deslorelin-bound proteins in rabbits is captured in 96-well plates by antibodies. A biotinylated tracer along with varying concentrations of unlabeled deslorelin-related protein standards compete for specific binding to the antiserum. Captured biotinylated tracer is subsequently bound by streptavidin-conjugated horseradish peroxidase which produces a soluble colored product after a substrate is added. By measuring the optical density at 450 nm, the deslorelin concentration is estimated from the resultant calibration curve with a limit of detection of 0.01 ng/mL [14].

Sakamoto et al. [15] have discussed the disadvantages of ELISAs which include high possibility of false negatives or positives and high dependence on antibody stability. Furthermore, uncertainty of the structure of ELISA antibodies arises on batch-to-batch comparison, making it difficult to eliminate the uncertainty factors of deslorelin ELISA using a uniform uncertainty estimation equation [16].

**Figure 2:**
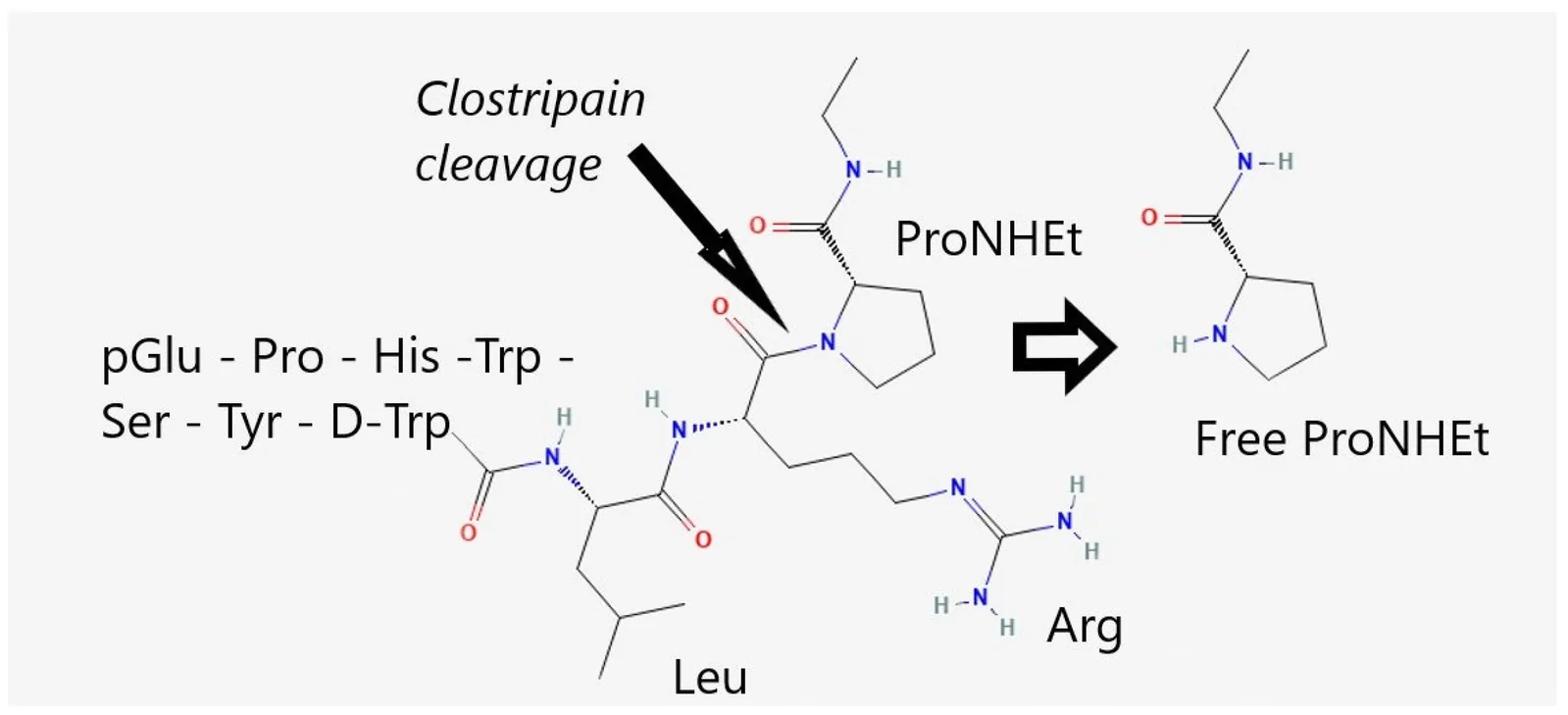
Structures and reaction sequence of the studied compounds. Arrow indicates the chemical bond located at the C-terminal side (carboxyl side) of the arginine residue cleaved by clostripain digestion. The enzymatic reaction releases N-ethyl-2-pyrrolidine carboxamide (ProNHEt), which can be released from deslorelin and similar synthetic gonadotropins by clostripain.

Deslorelin stability has been studied on reversed-phase high-performance liquid chromatography (HPLC) and liquid secondary ionization mass spectrometry (LSIMS), but the methods are nonspecific and insensitive [17]. Recent methods developed by Rooney et al. [18] with intact deslorelin measured on high-sensitivity ultraperformance liquid chromatography–tandem mass spectrometry (UPLC-MS/MS) have enabled determination to a quoted limit of detection of 8 pg/mL. This method has proven valuable in determining caprine pharmacokinetics of deslorelin. Its only disadvantage is its restriction to intact GnRH analog structures such as those of deslorelin or triptorelin. This is significant in that deslorelin can degrade in vivo to metabolites that often bear hormonal activity [19,20,21]. To improve the diagnosis of the hormonal status of animals after long term implantation of deslorelin, our work aimed to develop a novel method to precisely and accurately identify and quantify deslorelin and its breakdown products that bore the engineered terminal amino acid ProNHEt in animal serum by GC-MS/MS, with a detection limit of 0.6 ppb. To achieve this objective, our lab proposed enzymatic digestion of deslorelin into fragments with release of ProNHEt, which would be unique and measurable by GC-MS/MS. Upon studying the peptide sequence of deslorelin, we proposed an enzymatic cleavage of deslorelin using an arginine carboxypeptidase, which would release the carboxy terminal ProNHEt structure (Figure 2), as a surrogate marker for deslorelin. Clostripain, isolated from the culture of the anaerobic bacterium *Clostridium histolyticum*, has an apparent molecular weight of 50,000 Da and it exhibits the required proteolytic ability [22], i.e., it cleaves specifically after the arginine bond at the carboxy terminus of deslorelin [23,24].

The digested moiety of deslorelin, ProNHEt, can be extracted from digested serum and reacted with N-tert-butyldimethylsilyl-N-methyltrifluoroacetamide (MTBSTFA) to give a tert-butyldimethylsilyl (tBuDMS) derivative prior to analysis on GC-MS/MS [25]. Detection of the ProNHEt-tBuDMS derivative has applicability to intact and degraded deslorelin as well as related GnRH analogs that bear the Arg-ProNHEt terminal sequence; refer to Figure 1 for five GnRH analogs that bear this terminal group. Simultaneous studies of deslorelin on a matrix-assisted laser desorption/ionization–time-of-flight (MALDI-TOF) mass spectrometer served as a trouble-shooting tool to study digestion completion and provide a greater understanding of factors influencing deslorelin stability.

## 2. Materials and Methods

### 2.1. Chemical and Material Sources

Deslorelin was obtained from Sigma-Aldrich (St. Louis, MO, USA) as a lyophilized powder, which was reconstituted in deionized water at 1 mg/mL and stored at 4 °C. Guidelines state that upon reconstitution deslorelin should be stored at 4 °C between 2 and 7 days and for future use <−18 °C [26]. Clostripain (≥50 units/mg solid) from *Clostridium histolyticum* was obtained from Alfa-Aesar (Ward Hill, MA, USA). ProNHEt (CAS # 1060817-56-2) as its HCl salt, (2S)-N-ethyl-2-pyrrolidinecarboxamide hydrochloride (1:1), was obtained from Bachem (Vista, CA, USA) and dissolved in acetonitrile at 1 mg/mL and stored at −20 °C. Additional chemicals from Sigma included calcium chloride and DL-dithiothreitol (dissolved in deionized water and stored at 4 °C). The derivatizing agent labeled as MTBSTFA + 1% TBDMCS (N-tert-butyldimethylsilyl-N-methyltrifluoroacetamide with 1% tert-butyldimethylchlorosilane) was also from Sigma. Deionized water was prepared with a MilliQ filter from Millipore, Burlington, MA, USA. Acetonitrile (UV-grade) was from Honeywell–Burdick & Jackson, Muskegon, MI, USA. Screwtop autosampler vials and 250 μL conical glass inserts equipped with Teflon-lined caps were from Agilent Technologies (Santa Clara, CA, USA).

### 2.2. Handling of Clostripain

Clostripain induces selective hydrolysis of Arg bonds, with Lys bonds cleaved at a lower rate [27]. Its activity depends upon a cysteine thiol group, so a reducing agent such as DTT is included in the assay buffer, and there is an absolute requirement for Ca^2+^ ions at 10 mM. The pH optimum for the hydrolytic reactions is 7.2–7.8. Potent inhibitors of clostripain include oxidizing agents, thiol-blocking agents, Co^2+^, Cu^2+^, Cd^2+^ and heavy metal ions [28]. An enzyme storage buffer consists of 20 mM sodium phosphate buffer, pH 7.5, 2.5 mM dithiothreitol (DTT), and 1 mM CaCl_2_ in deionized water. Components were prepared individually at 10× concentrations and diluted to give the listed mixture prior to use. The 10× sodium phosphate buffer was created by preparing 0.2 M dibasic sodium phosphate (Na_2_HPO_4_) and 0.2 M monobasic sodium phosphate (NaH_2_PO_4_) and mixing them together until pH 7.5 was achieved, typically in a ratio of roughly 1 part monobasic: 4 parts dibasic. Clostripain was available as ≥50 units/mg lyophilized powder of 250 total units, thus about 12.5 mg. For enzyme activation, 1.0 mg was dissolved in 1.0 mL of storage buffer and incubated 17–24 h at 4 °C [27] for a 1 mg/mL solution. This was diluted 1:10 in storage buffer, and individual 1 mL aliquots at 0.1 mg/mL were stored in microcentrifuge tubes at −20 °C.

### 2.3. Clostripain Reaction

Serum containing the target deslorelin contains at least 5 mg/mL protein and the enzyme:substrate ratio on a *w*/*w* basis should be at least 1:100 [29]. Stock components for the reaction included 200 mM sodium phosphate buffer, pH 7.5, 125 mM dithiothreitol (DTT), 50 mM CaCl_2_, each in deionized water. For reactions for proof of concept, the following were added in the given order in microcentrifuge tubes: (1) 50 µL deslorelin (1 mg/mL), (2) 80 µL Bovine Serum Control or other serum sample; (3) 75 µL deionized water; (4) 10 µL 50 mM CaCl_2_; (5) 10 µL 125 mM DTT; (6) 25 µL 200 mM phosphate buffer, pH 7.4; (7) 250 µL clostripain (0.1 mg/mL, thawed briefly on ice before use). The mixture was vortex-mixed gently, then placed at 25 °C for incubation overnight (≥16 h). The resultant mixture would have about 0.25 U clostripain per mg protein.

### 2.4. GC-MS/MS

Triple quadrupole GC-MS/MS analyses for MTBSTFA-derivatized ProNHEt and deslorelin cleavage products were performed on the Agilent 7890A/7000 instrument (Santa Clara, CA, USA) equipped with a Phenomenex (Torrance, CA, USA) ZB-CL Pesticides-1 column of dimensions 15 m × 250 μm × 0.25 μm hooked by a backflush T to a 30 m column of the same type. Helium flow rates were 1.2 and 1.4 mL/min, respectively, for the 1st and 2nd columns. The Multi-mode inlet of the GC-MS/MS was set at 210 °C and was run in splitless mode. The gas chromatograph oven temperature program was as follows: 110 °C held for 2 min; then 21 °C/min to 180 °C held for 0.5 min; then 15 °C/min to 250 °C held for 1 min; then 24 °C/min to 315 °C held for 14 min, for a total run time of 28.0 min. The injection volume was 1 μL. He Quench Gas flowed at 2.25 mL/min, whereas N_2_ Collision Gas flowed at 1.0 mL/min. The gain setting on the MS was 30. In studies where full-scan MS analysis was required, the acquisition range was from *m*/*z* 40 to 600. Multiple Reaction Monitoring (MRM) methodologies appropriate to GC-MS/MS were developed by a combination of initial product ion studies followed by optimization of fragment ion responses. MRM settings were optimized by acquiring data at collision energies of 1, 2, 5, 10, 15, 20, 25, 30, 35, 40 and 50 eV and picking optimum values. Data analysis proceeded with the use of Agilent Mass Hunter Workstation software, version 10.2.

### 2.5. Sample Derivatization

MTBSTFA derivatives offer the advantages of being more stable and less moisture sensitive than those created by lower molecular weight reagents such as N,O-bis(trimethylsilyl)trifluoroacetamide (BSTFA). Derivatization of this type replaces active hydrogens on OH, NH, NH_2_, and SH polar functional groups with a nonpolar moiety. Dried extracts and analytical standards were derivatized by addition of 50 μL of MTBSTFA + 1% TBDMS and 50 μL of acetonitrile to dried tubes containing extract residues. The tubes were shaken on a rotary shaker, transferred to autosampler vials equipped with inserts, sealed and subsequently incubated at 70 °C for 60 min to induce the derivatization reaction to occur [30]. If not analyzed by GC-MS/MS directly, vials were stored in a refrigerator and analyzed within the next 24 h.

### 2.6. Mass Spectral Interpretation

Where necessary, fragmentation characteristics of derivatives were studied with the aid of Mass Spec Calculator Pro (Quadtech Associates, https://quadtechassociates.com/ (Accessed 28 August 2026)).

### 2.7. Matrix-Assisted Laser Desorption/Ionization–Time-of-Flight (MALDI-TOF) Analysis

The sample (1.5 µL) was spotted on individual wells of a stainless steel MALDI target plate (Bruker Daltonics, Billerica, MA, USA), dried and overlaid with 2 µL α-cyano-4-hydroxycinnamic acid matrix solution (Bruker Daltonics), following the manufacturer’s recommended protocol. Peptide spectra were captured using the Microflex LT MALDI-TOF MS (Bruker Daltonics) following the manufacturer’s recommended protocols. For every run the mass spectrometer was calibrated for molecular weights with a range of 0.75–3.15 kDa prior to sample testing using Peptide Calibration Standard II (Bruker Daltonics) as per the manufacturer’s recommendations (available at https://store-stage.bruker.com/products/peptide-calibration-standard-ii, accessed 15 October 2026). The spectra were captured in positive linear mode in a mass range of 760–3300 Da, with laser frequency of 200 Hz (IS1: 10 kV; IS2: 8.99 kV; lens: 3 kV; extraction delay time: 140 ns). Spectra were manually acquired, and the mean of 3 spectra (from the same well/sample) was used for further analysis.

### 2.8. Deproteinization and Derivatization

Extraction was initially based on methods of Pérez-Palacios et al. [30]. That group deproteinized samples by combining 250 μL of acetonitrile with 100 μL of extracts in micro test tubes, centrifuging at 10,000 rpm for 3 min, then transferring 100 μL of the supernatant to heat-resistant tubes for evaporation under a stream of nitrogen. The evolved method for deslorelin digests in serum required addition of 4 mL acetonitrile to 1 mL spiked or digested serum (digestion mixtures diluted with deionized water to 1 mL total) in 50 mL centrifuge tubes, adding at least 1.5 g sodium chloride, vortexing for at least 30 s, centrifuging 5 min at 3000 rpm in the Beckman tabletop centrifuge at room temperature, and transferring the top acetonitrile layer to a clean tube. An additional 4 mL aliquot of acetonitrile was then added to the original tube which was then vortexed at least 30 s, centrifuged as before, and the top acetonitrile phase combined with the initial extract. This combined phase was then passed through a funnel containing anhydrous sodium sulfate, rinsed with one mL of acetonitrile and combined with 20 μL N,N-dimethylformamide as a keeper solvent. This suspension was dried under a stream of nitrogen to near dryness and resuspended in 0.5 mL acetonitrile. Finally, 50 μL of MTBSTFA and 50 μL of the final acetonitrile suspension were added to new tubes, which were shaken and subsequently incubated at 100 °C for 60 min to induce the derivatization reaction to occur. Then, the tubes were stored in a refrigerator and analyzed by GC-MS within the next 24 h.

### 2.9. Structural Studies

Deslorelin was modeled in silico by means of Hyperchem (http://www.hypercubeusa.com/ (Accessed 28 August 2026)), version 8.0.10. The molecular mechanics mode was applied along with the Amber force field.

## 3. Results

### 3.1. Establishment of an MRM Detection Method on GC-MS/MS

**Figure 3:**
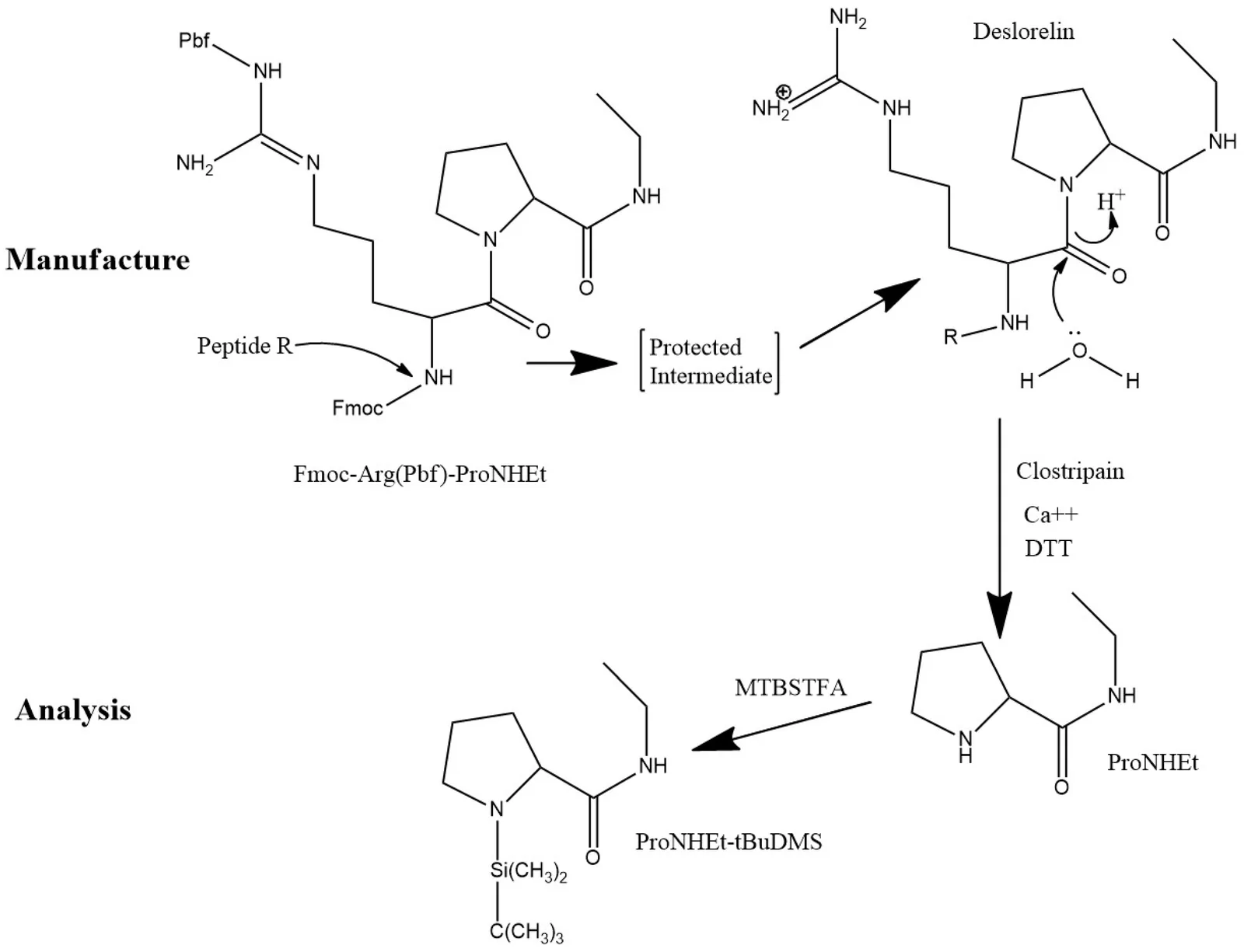
Overall pathway to the deslorelin detection product. One synthetic building block used by the pharmaceutical industry to gonadotropins involves Fmoc-Arg(Pbf)-ProNHEt (left) coupled with an appropriate peptide precursor R. Protecting groups are Fmoc = 9-fluorenylmethyloxycarbonyl and Pbf = 2,2,4,6,7-pentamethyldihydrobenzofuran-5-sulfonyl. The product could be deslorelin or leuprorelin, for example, depending on the structural sequence of the added Peptide R. Clostripain digestion in the analytical lab with cofactors calcium and dithiothreitol (DTT) releases ProNHEt (N-ethyl-2-pyrrolidinecarboxamide) which is subsequently reacted with MTBSTFA (with catalyst 1% TBDMSCl) to create the ProNHEt-tBuDMS derivative detectable by GC-MS/MS. Straight arrows represent the overall transformation of reactants into products; slightly curved arrows display the introduction of a chemical reagent; small curved arrows depict the microscopic movement of electrons within a specific step of a reaction mechanism.

The overall pathway from introduction of ProNHEt to deslorelin during drug manufacturing to its digestion and derivatization in the lab is shown in Figure 3. In brief, the protecting groups 9-fluorenylmethyloxycarbonyl and 2,2,4,6,7-pentamethyldihydrobenzofuran-5-sulfonyl are removed from an intermediate to provide deslorelin. For analysis in the lab, ProNHEt is cleaved from deslorelin with clostripain, its catalyst Ca^2+^ and its sulfhydryl protecting agent DTT. ProNHEt is finally derivatized with MTBSTFA to provide a t-butyldimethylsilyl (tBuDMS) derivative which is the quantitative marker examined by GC-MS/MS for deslorelin.

**Figure 4:**
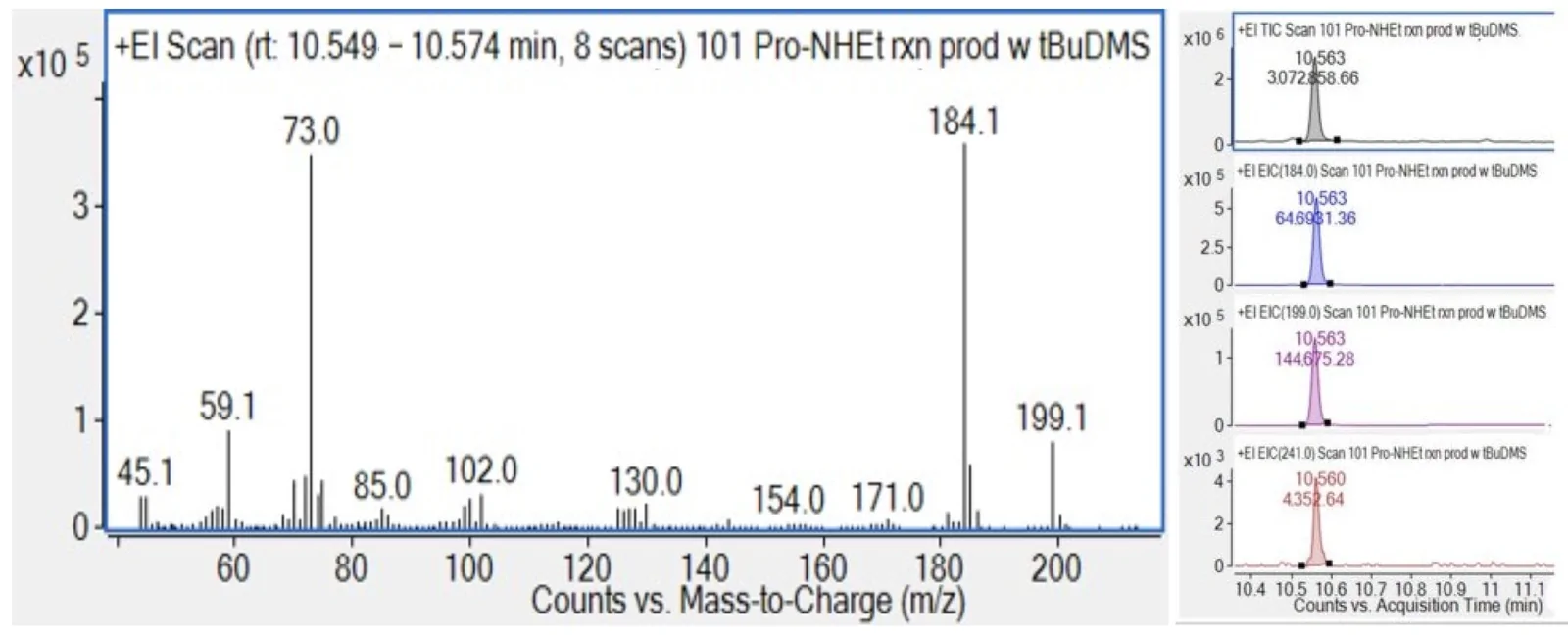
(**Left**) Pro-NHEt-tBuDMS EI-mass spectrum, as run on the GC-MS/MS. (**Right**) Ion chromatography of key *m*/*z* values in the Pro-NHEt-tBuDMS spectrum, namely, top-to-bottom TIC (Total Ion Chromatogram), *m*/*z* 184, 199, and 241.

**Figure 5:**
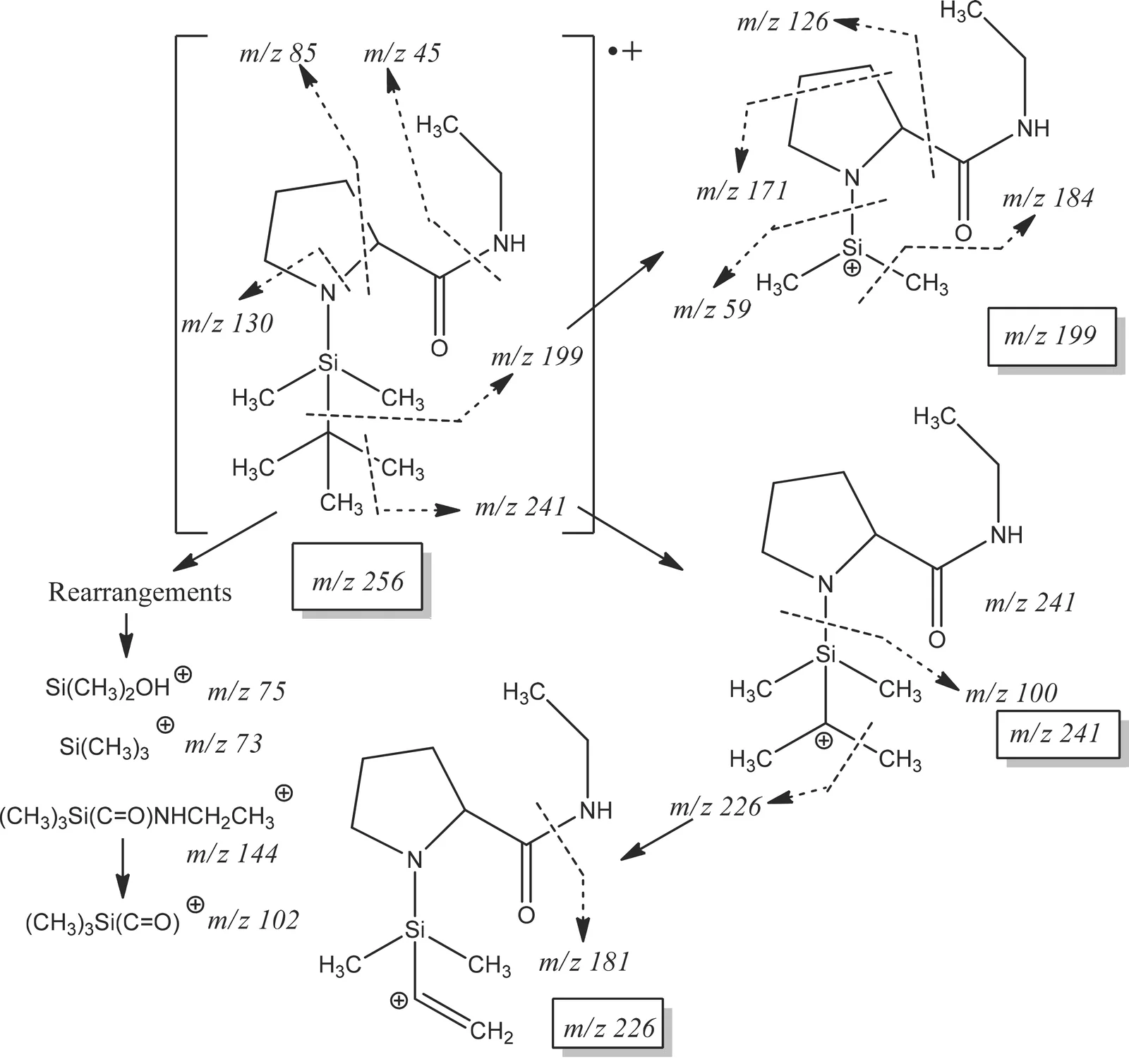
Proposed electron impact fragmentations to account for the mass spectrum of the ProNHEt-tBuDMS derivative, starting as an anticipated *m*/*z* 256 molecular radical cation. Dotted arrows show proposed fragmentation events, while solid arrows show pathways to resultant products.

The full-scan electron-impact mass spectrum of ProNHEt-tBuDMS is shown in Figure 4 left. Ion chromatography (Figure 4 right) demonstrated that fragments *m*/*z* 184, 199 and 241 correspond to the principal peak shown in the total ion chromatogram (TIC), making *m*/*z* 241 the highest visible peak in the spectrum of what would be a 256 m.w. compound. Figure 5 shows an interpretation of the fragmentation of ProNHEt-tBuDMS from a proposed molecular ion of *m*/*z* 256 with key intermediates *m*/*z* 241 and 199 arising from loss of a methyl or loss of a t-butyl group, respectively.

**Figure 6:**
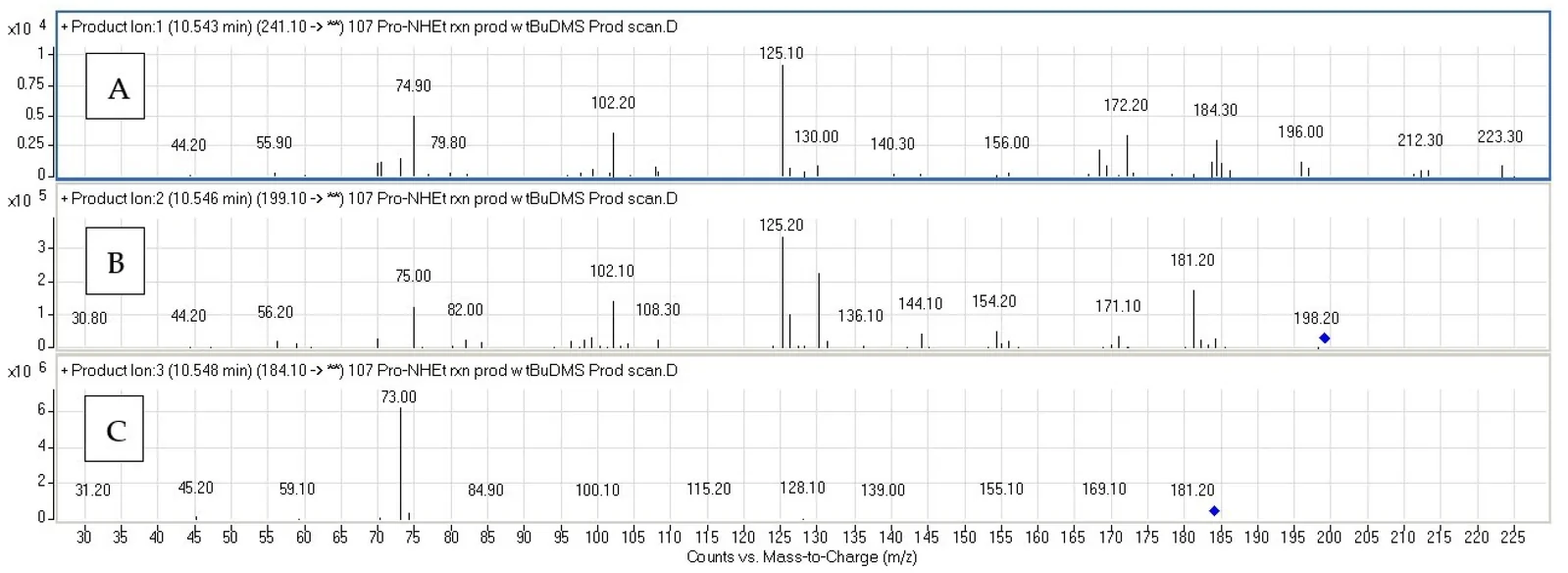
GC-MS/MS study of ProNHEt-tBuDMS by product ion study. Shown are product ion mass spectra derived from the principal chromatographic peak at 10.54-min. (**A**), (**B**) and (**C**) show product ion mass spectra from *m*/*z* 241, 199, and 184, respectively, acquired at collision energy 15 eV. Symbol ** indicates any *m*/*z* values in the scan range. Blue diamonds 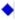 represent the location of the parent *m*/*z* value, where visible.

Product ions were screened from the principal *m*/*z* ions 184, 199 and 241, and the resultant mass spectra are shown in Figure 6. Collision energies (CEs) were optimized for candidate Multiple Reaction Monitoring fragmentations by recording peak areas for CE values of 1, 2, 5, 10, 15, 20, 25, 30, 40 and 50 eV, and the resultant relationships are shown graphically in Supplement Figure S1.

**Figure 7:**
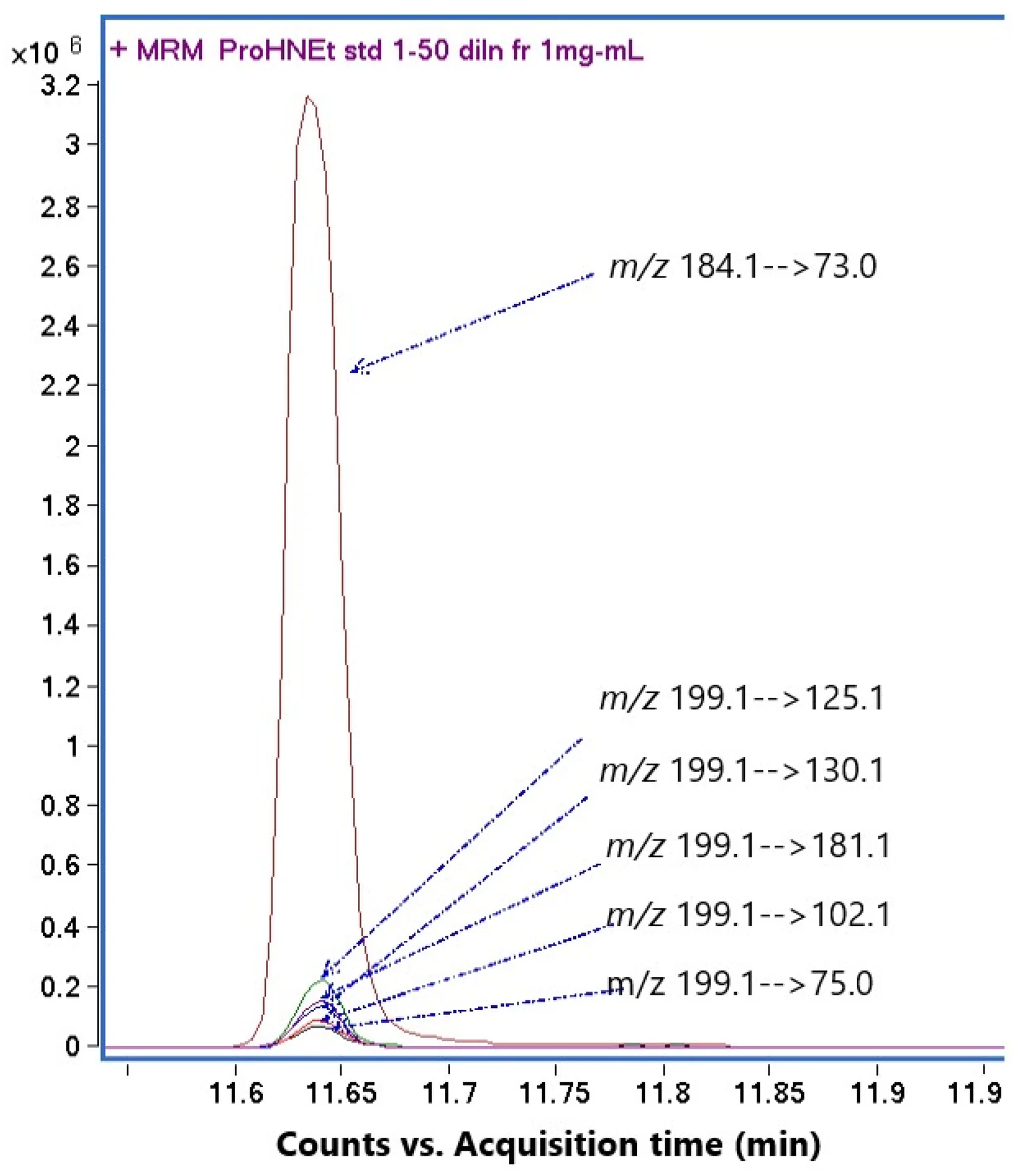
MRM ion chromatography of Pro-NHEt tBuDMS derivative by the CE-optimized method with nested individual peaks as labeled. Cleavage of *m*/*z* 184 to release a *m*/*z* 73 fragment is the most sensitive transition for quantitation of ProNHEt as the tBuDMS derivative.

Table 1 summarizes the precursor → product ion *m*/*z* values comprising ProNHEt-tBuDMS MRM settings and their respective collision energies. Figure 7 shows ion chromatography for selected MRMs indicating that *m*/*z* 184→73 predominates with diminishing peak sizes for MRMs originating from *m*/*z* 199 as shown. Figure S2 in the Supplementary Materials shows the ratio of potential qualifier MRMs to the *m*/*z* 184→73 quantifier MRM indicating that all wound up at less than 10% relative area.

### 3.2. Optimization of Chromatographic Settings for High Sensitivity

MTBSTFA reactions in the VDL Toxicology lab have typically been performed at 60 or 70 °C. Figure S3 in the Supplementary Materials shows that 60 °C works better, providing ~3–4-fold better area responses than those at 70 °C. This study was performed in duplicate giving identical results at the preferable temperature.

GC-MS parameters were optimized for maximum sensitivity, and results are shown in Supplement Figure S4. Specifically, the nitrogen flow to the collision cell, effect of gain in the MS detector, GC inlet temperature and initial oven temperature for chromatography were individually studied as shown. The results indicate that (1) nitrogen flow appeared optimal at 1 mL/min; (2) gain response increased quite linearly across the Gain 10–40 range as hoped; (3) inlet temperature was good at 210 °C, and higher or lower temperatures offered no advantages; and (4) an initial oven temperature of 110 °C appeared to be optimal. Linearity of response depending on concentration was determined across the 10–10,000 ppb (ng/mL) range at Gain = 30 and is shown in Supplement Figure S5. The coefficient of determination R^2^ was 0.9989 demonstrating good linear regression.

### 3.3. Determination of ProNHEt Limits of Detection and Quantitation

Limits of detection (LODs) and quantitation (LOQs) can be determined from signal-to-noise (S/N) ratios measured for a series of standard concentrations [31]. The S/N value of 3 then corresponds to the LOD and the value of 10 corresponds to LOQ. Agilent Mass Hunter software determines S/N ratios during quantitative analysis by application of an RMS (root mean square) algorithm with an applied noise standard deviation multiplier setting of 5.0. The determined S/N ratios were applied to standards of 0, 500 and 2500 ppb in three instances. Application of this approach gave values of LOD = 0.15 ± 0.08 ppb and LOQ = 0.60 ± 0.3 ppb. Area ratios of qualifier MRMs were determined for concentrations of 0.1, 1.0, 10, 100, and 1000 ppb. Ratios were quite consistent in the range 1.0–1000 ppb, but rose dramatically for 0.1 ppb, which is consistent with the calculated LOQ; see illustration in Figure S6 in the Supplementary Materials. Figure S7 shows the quantifier MRM chromatographic peaks for 0.1 and 1.0 ppb; their S/N ratios measured manually by area are 33.7 and 54.7, respectively. Given that these values are in excess of 10, it may be possible to lower sensitivity, for example, by larger injection volumes, but any lower LOQ values would need to be supported by consistent area ratios.

### 3.4. Additional Validation Determinations for ProNHEt

Linearity was assessed by studying eight standard curves arrayed over the range of standards 50–10,000 ppb. Regression provided an average coefficient of determination R^2^ of 0.99605 ± 0.00331. Refit values were determined for three of the standard curves and residuals were calculated as nominal value minus refit value. A plot of the residuals versus nominal value is given in Supplement Figure S8; the result shows values scattered around the x-axis with no significant deviation with change in concentration. Interday precision was determined by collecting refit values on eight standard curves, calculating average results and standard deviations, and calculating %RDS (relative standard deviation) across the standard range 50–10,000 ppb. Results listed in Supplement Table S1 show %RSD results of 4.5% for 50 ppb, 6.4% for 500 ppb, 8.8% for 2500 ppb, 7.1% for 5000 ppb, and 1.0% for 10,000 ppb. Intraday precision was calculated similarly for three standard curves generated across a single day and the results are listed in Supplement Table S2. This shows %RSD results of 4.5% for 50 ppb, 6.6% for 500 ppb, 3.0% for 2500 ppb, 3.9% for 5000 ppb, and 0.3% for 10,000 ppb. Specificity was assured by inclusion of qualifier MRMs, but these can vary to a certain degree depending on changes in instrument performance day-to-day. The results for 24 measurements on a single day are given in Supplement Table S3 and provide the results: *m*/*z* 199.1 → 75.0, 49.2% ± 3.91%; *m*/*z* 199.1 → 102.1, 53.1% ± 4.03%; *m*/*z* 199.1 → 130.0, 56.7 ± 3.31%; *m*/*z* 199.1 → 181.2, 61.3% ± 1.17%. Instrumental carryover was assessed on the basis of response of blank samples run after the highest calibrator of 10,000 ppb; this gave values; seven determinations gave average carryover of 0.0055% ± 0.012%. Standard curve accuracy was determined on the basis of refit values for standards on their respective curves, which gave values of 103.5% (50 ppb), 95.2% (500 ppb), 101.7% (2500 ppb), 102.0% (5000 ppb) and 99.4% (10,000 ppb). Retention times can vary slightly from day to day depending on column maintenance and inlet cleanliness. Nevertheless, retention times during a given run appear stable. Measurement across 36 GC-MS/MS scans provided an average retention time for ProNHEt-tBuDMS of 10.894 ± 0.198 min with a corresponding %RSD of 1.8%. Recovery was determined by development of a standard curve across concentrations 500–10,000 ppb, then applying the results from serum-extracted standards at the same concentrations, as listed in Supplement Table S4. The average recovery of 35% is arguably low and could be improved upon by considering solid-phase extraction approaches. Styrene-divinylbenzene (PS-DVB) resins are widely used for the solid-phase extraction (SPE) of amino acids and related compounds. For instance, sulfonated poly(styrene-divinylbenzene) modified with amine functional groups has been applied to the determination of the carbamate fungicide carbendazim in complex matrices like apple and wine samples using pipette-tip SPE [32].

### 3.5. Consideration of an Internal Standard

An internal standard can perform two or more roles, the most significant being a reference point in standard curve formation for averaging out minor differences between adjacent standards or samples with regards to extractions or instrument performance. A deuterated or ^13^C-labeled compound would involve a process of isotope dilution as one proceeds through the range of increasing standard concentrations; however, its creation as a specialized standard would be cost-prohibitive. One additional value of an internal standard is to provide an independent measure of extraction efficiency with the hoped-for goal of perfect uniformity between responses in unextracted standards and those in extracted matrices demonstrating lack of strong matrix interference. An internal standard was not used in the work described here, but a suggested approach would be the use of Triptorelin at a point prior to clostripain digestion. The product would be the amide of the dipeptide Pro-Gly. Pro-Gly can be purchased for ~$400/g and amide formation would be accelerated by use of dicyclohexylcarbodiimide as a coupling agent [33]. The tBuDMS derivative is calculated to have a molecular weight of 271.2 which should make it readily distinguishable from the ProNHEt molecular ion at 256.2.

### 3.6. Optimization of the Clostripain Cleavage Reaction

**Figure 8:**
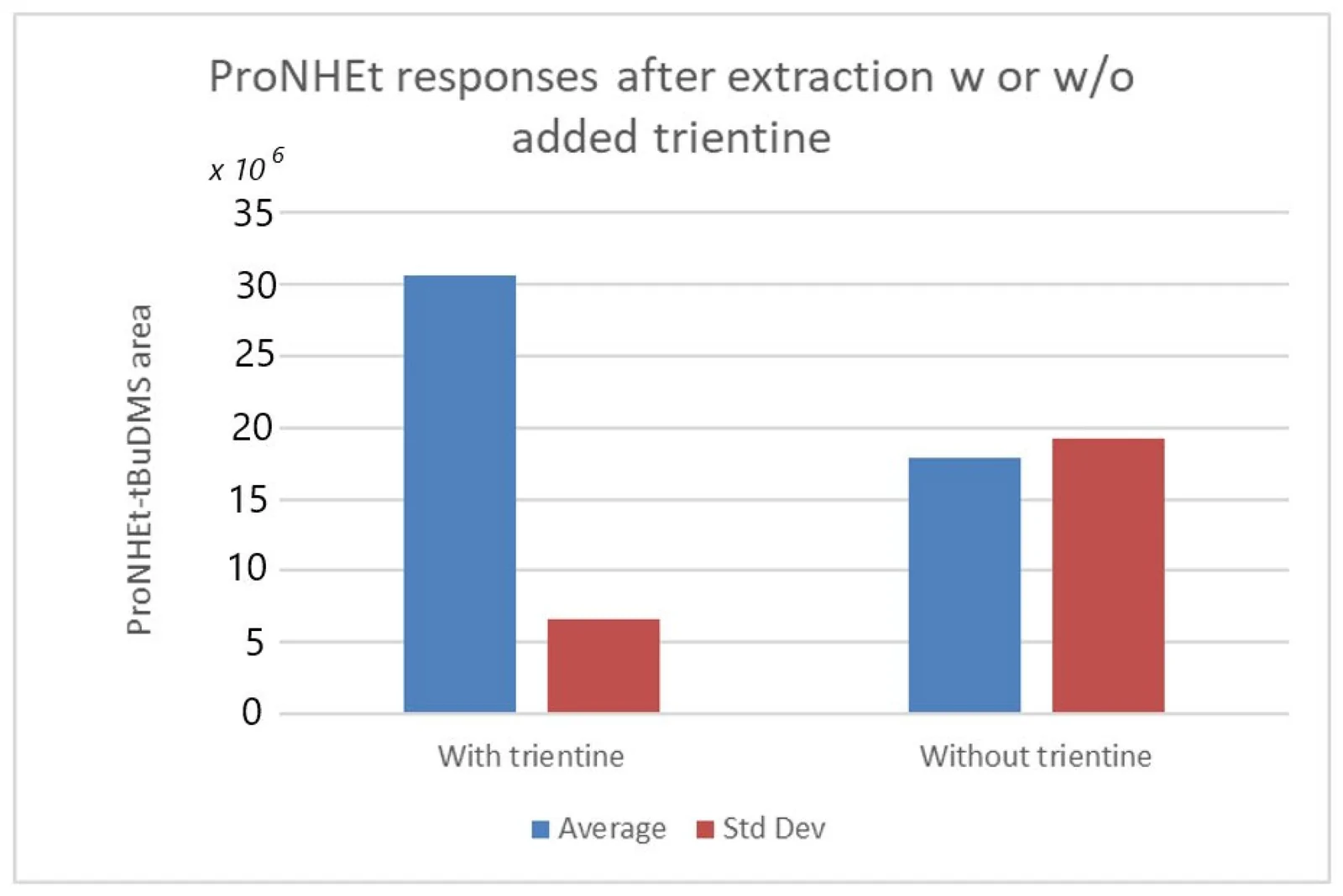
ProNHEt-tBuDMS responses with and without added trientine during the clostripain cleavage reaction. The graphical display shows greater average response and lower standard deviation with use of trientine. Average recovery with deslorelin alone: 30.1% +/− 12.1%; average recovery with trientine added: 42.5% +/− 3.3%.

The clostripain enzyme requires calcium as a necessary catalyst but at the same time is sensitive to the presence of copper. Trientine is a chelating agent that is highly selective for copper and to a lesser extent iron and zinc [34] and was thus added to incubations of deslorelin with the enzyme for determination of ProNHEt responsiveness. Results of five experiments with and five without added trientine are shown in Supplement Figure S9. Figure 8 summarizes greater average responsiveness and lower standard deviation upon use of the chelating agent, likely owing to protection against interfering cations. This therefore represents an increase in clostripain enzyme efficiency. It would be ideal to carry out this determination at multiple deslorelin concentrations to arrive at an idea of method robustness.

It was deemed important to maintain the stability of the target compound deslorelin, its product ProNHEt and the clostripain enzyme to oxidizing conditions, e.g., from aqueous solubilized oxygen. With this in mind, the antioxidant butylated hydroxytoluene (BHT) was added at the same concentration to the standard reaction mix under various conditions as shown in Supplement Figure S10. In each case BHT improved the total yield of ProNHEt as judged by the area counts for the *m*/*z* 184→73 MRM fragmentation. It did not offer any improvement in the event of UV exposure.

An initial concern was whether the target matrix of serum would have an inhibitory effect on the clostripain reaction, owing not only to introduction of inhibitory cations such as copper which exists in human serum in the range 60–160 μg/dL [35], but also owing to introduction of additional proteins acting as competitors for the active enzyme. It was reassuring to discover that the opposite was the case, namely, that introduction of 25, 50 or 75 μL of bovine serum increased the area counts for ProNHEt with near-linear responsiveness (Supplement Figure S11). This implies the existence of a protective effect of serum on the cleavage reaction, encouraging in turn the addition of as much target matrix into the reaction as possible.

Nishimura et al. [29] listed 24 °C as the incubation temperature used for clostripain digestion, whereas Labrou & Rigden [23] list it as 25 °C. Optimal retrieval of ProNHEt was studied at 18, 25, 32 and 37 °C, and the optimal temperature is in the vicinity of 32 °C as shown graphically in Supplement Figure S12.

### 3.7. Improvements in ProNHEt Extraction

There was a concern that ordinary lab procedures could result in losses of ProNHEt during the nitrogen evaporation step due to amine volatility [36]. One means of preventing such losses due to compound volatility was the simple matter of adding a high-boiling solvent to the final solvent mixture, such that N,N-dimethylformamide was added to provide a liquid medium in the residue. Another means would be conversion to a HCl salt [37]. Figure S13 in the Supplement shows the effect of addition of 150 μL 0.1 M HCl to the final acetonitrile extract (~8 mL) before nitrogen evaporation. The 150 μL volume afforded considerable improvement over 0 or 300 μL addition, considered to be the likely consequence of conversion of the ProNHEt secondary amine to a HCl salt.

Another approach used in the MSU VDL Toxicology section during Quechers-type extraction [38] involves the introduction of a freezing step of the acetonitrile phase (1 h at −20 °C) prior to the evaporation step. Inclusion of such a step roughly doubled the recovery of ProNHEt owing to removal of a fat layer that could bind hydrophobic molecules.

### 3.8. Proof of Concept: Enzymatic Release of ProNHEt

**Figure 9:**
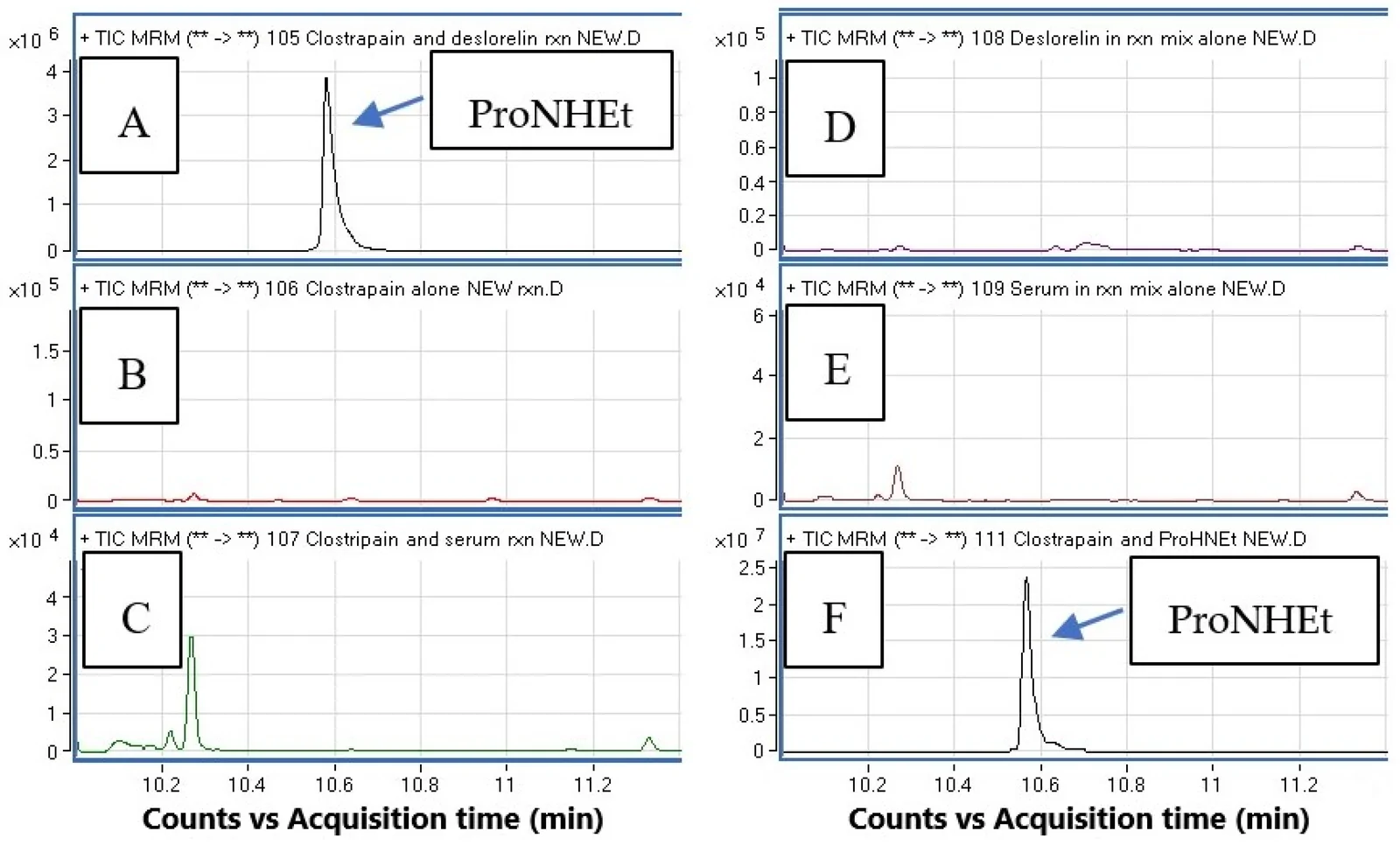
GC-MS/MS analysis of various deslorelin/clostripain/ProNHEt combinations to discover the presence of peaks coeluting with ProNHEt at 10.6 min retention time. Recipes are shown in Supplement Table S5, and expectations are listed in Table 2. Combinations include (**A**) clostripain and deslorelin; (**B**) clostripain alone; (**C**) clostripain and serum; (**D**) deslorelin alone; (**E**) serum alone; (**F**) clostripain and ProNHEt. ProNHEt is expected only in (**A**,**F**). One experiment is shown here, but the results were repeated in a second trial. The expression **->** indicates any of the MRM precursor ions fragmenting to their respective product ions.

A concern was whether enzymatic digestion of the target matrix of serum would release compound(s) that gave false positives for the presence of ProNHEt. A series of digestions were created with appropriate controls as defined by the recipes of Table S5 of the Supplementary Materials. These included: (1) Clostripain combined with the target deslorelin; (2) Clostripain alone as a control on any self-digestion; (3) Clostripain combined with serum in case serum proteins could generate ProNHEt or its possible mimics; (4) Deslorelin itself in case of spontaneous degradation with release of ProNHEt; (5) serum alone in case it contained ProNHEt mimics without the need for enzymatic treatment; and (6) Clostripain plus ProNHEt with the concern that the enzyme could have some further effects on the target molecule. Table 2 lists the expectations of the presence of ProNHEt, specifically in tests (1) and (6) of the above list, and its absence in the rest. Figure 9 shows the results of these tests in order # A–F, and every test met expectations. This examination was repeated successfully illustrating proof of the overall concept and the likely lack of interfering components. Note that deslorelin was introduced at a final concentration of 100 μg/mL in the digestion series, well above the effective concentration of deslorelin in animal serum of roughly 25 ng/mL [18].

### 3.9. Structural Studies on Target Deslorelin

**Figure 10:**
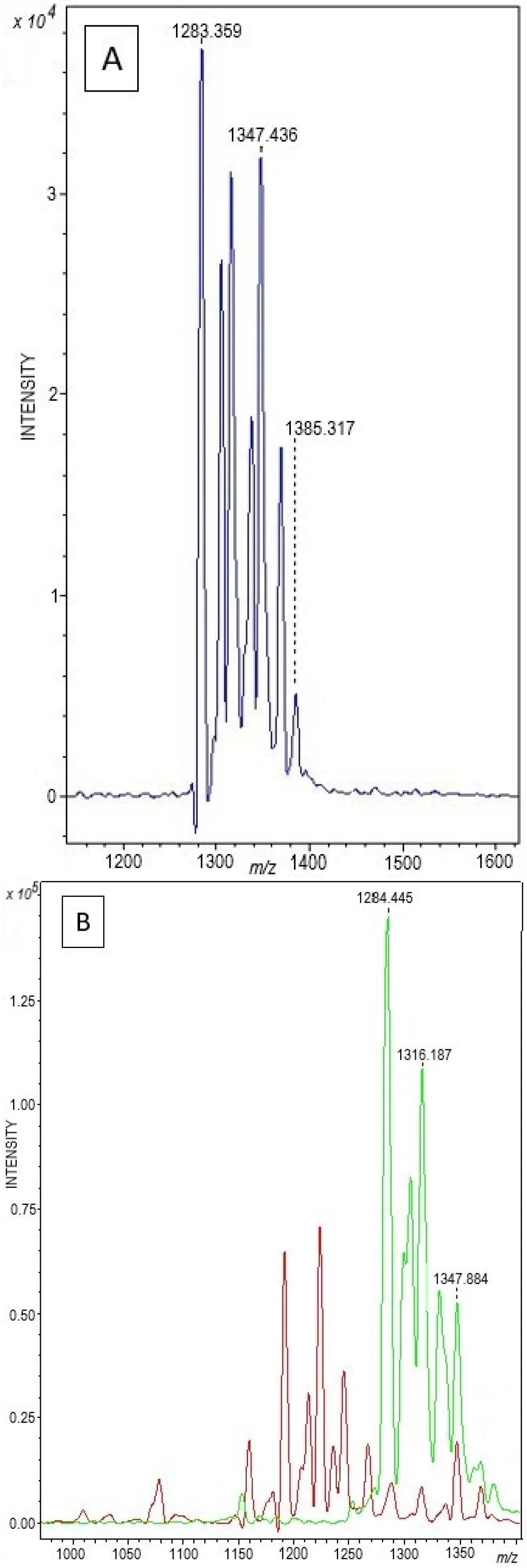
Deslorelin 10 ppm in filtered serum examined by MALDI. (**A**) Direct examination of Deslorelin; (**B**) overlap of unaltered deslorelin (green) with clostripain-digested deslorelin (red).

Deslorelin was studied by MALDI mass spectrometry to better understand the target compound and its enzymatic digestion. Figure 10A shows the resultant mass spectrum and positive identification of deslorelin required a slight shift in *m*/*z* values owing to minor inaccuracies in the MALDI spectrometer. Specifically, acquisition of the MALDI calibration standards showed increasing errors at higher *m*/*z* values, as shown in Supplement Table S6, part A. Arrangement of the expected values as a function of the measured values provided a chart for correction of measured values as shown in Supplement Figure S14. Figure S14 also shows the accurate mass determination for formula C_64_H_83_N_17_O_12_ for deslorelin yielding an unprotonated value of 1281.6406. The Figure S14 chart in turn enabled adjustment of deslorelin values as shown for two examples in Supplement Table S6, part B. Thus, confusing *m*/*z* values of 1284.4 or 1283.4 are not in actuality +2 or +1 amu over the expected value of 1282.6, but are closer to the target value.

The Figure 10A spectrum of deslorelin revealed a surprising number of molecular components. Of nine principal components, one was deslorelin plus an acid-derived proton to give deslorelin + H^+^, but four more could be explained by addition of one, two or three atoms of oxygen to give oxidized deslorelin species. Deslorelin standards were admittedly not layered with inert gases such as argon or nitrogen during freezer or refrigerator storage which may have otherwise curtailed oxidation events. Amino acids susceptible to oxidation comprise ones that include the sulfur atom (methionine, cysteine) and ones with aromatic rings (histidine, tryptophan, tyrosine) as discussed in the scientific literature [39,40,41], and deslorelin includes the aromatic ring ones (his, trp x 2, tyr) as shown in Figure 2. Deslorelin also displayed several species that could be best explained as complexes with Cu cations. This is also not unexpected, given that copper binding is an evolutionarily ancient feature of GnRH-related peptides [42], where Cu can alter the structure and function of such neuropeptides, potentially enhancing their abilities to release luteinizing hormone (LH) and follicle-stimulating hormone (FSH), as reviewed by Peacey et al. [43]. This could be viewed as potentially problematic for deslorelin integrity in that transition metals often catalyze oxidation of aromatic rings, as reviewed by Wang & Xiao [44]. At the same time Cu could also be problematic for the proposed procedure, since Cu is also a known inhibitor of the clostripain enzyme. Fortunately for the proposed method, Cu(II) is generally thought to bind to the N-terminus of GnRH by coordinating between pGln (pyroglutamic acid or 5-oxoproline) and His residues [45].

**Figure 11:**
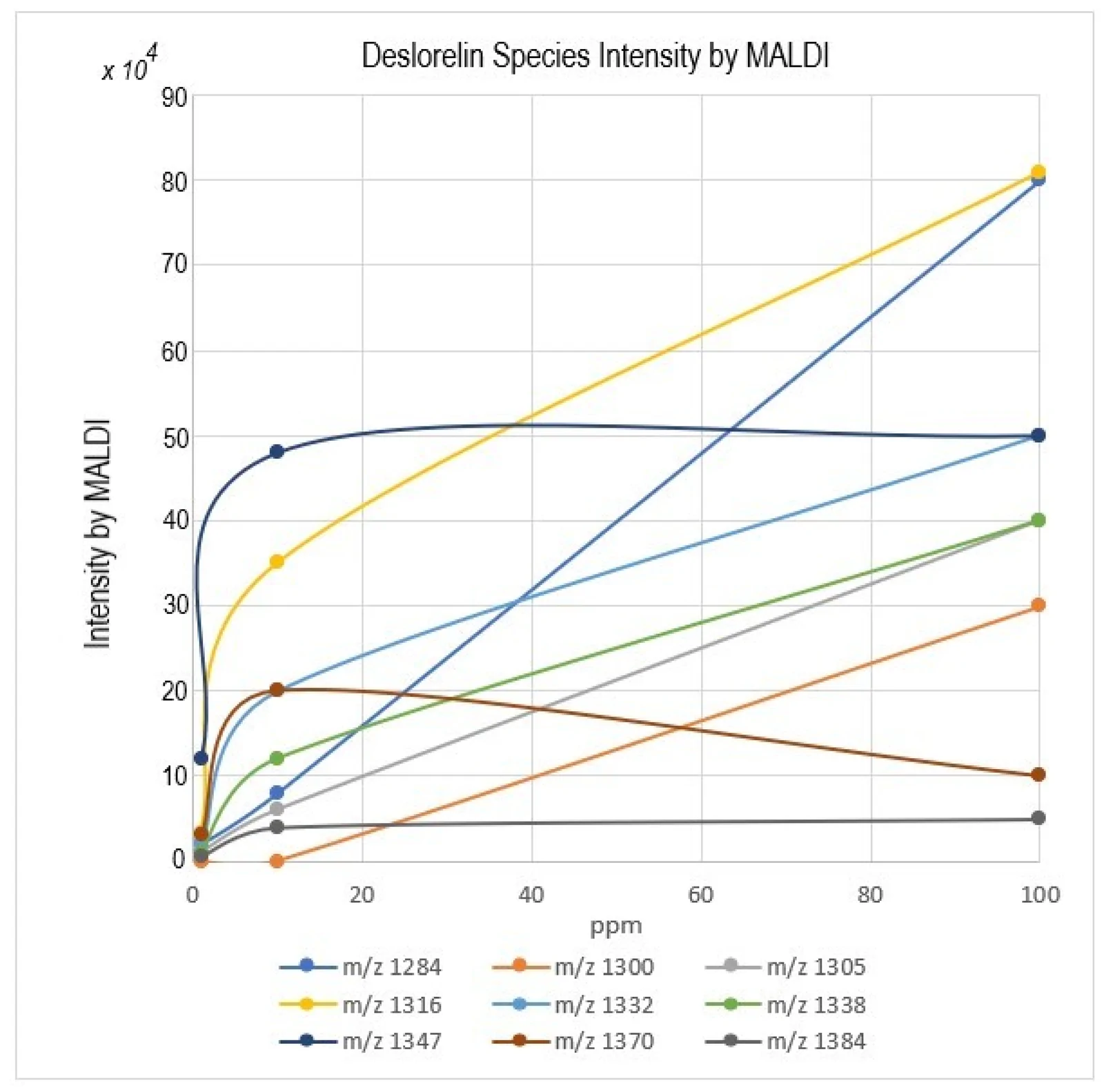
Variation in intensities of MALDI-detected deslorelin Species over the range of concentrations shown. The various species show increases, except for the putative Cu-bearing variants (*m*/z 1347, 1370 and 1384) which plateau or decrease with increasing deslorelin concentration. This was the result of a single experiment.

Figure 10B also shows a MALDI experiment in which deslorelin was examined both before and after digestion with clostripain. The impression is conveyed that the gamut of deslorelin peaks between *m*/*z* 1284 and 1384 are shifted to the left to a grouping between *m*/*z* 1159 and 1259 with a retention of the pattern of peaks. Cleavage as in Figure 2 to release ProNHEt would necessarily include addition of an OH to residual deslorelin to complete the hydrolysis reaction with formation of a terminal COOH group at the arginine residue. Table 3 lists the expected *m*/*z* values for such cleavage products and in nearly every case the corresponding product was found as expected. This provides support for the initial structural assignments thereby underscoring the retention of the oxidation and copper complexes in the cleavage products. However, a range of deslorelin concentrations were studied with resultant mass spectra shown in Figure S15 in the Supplementary Materials. As seen in Figure S16, responses for deslorelin as its + H^+^ and + Na^+^ positively-charged adducts were linear across the 1–100 ppm range. However, plotting intensities for all deslorelin species as shown in Figure 11 shows such linearity does not occur with the presumed Cu-bearing species. This implies that high concentrations of deslorelin were diluting out the available Cu(II) ions resulting in plateau or diminishing responses. The assigned structures of the MALDI-revealed deslorelin derivatives should be considered tentative and could be adjusted and/or proven if further studies were carried out. In addition to well-designed MALDI experiments, an important approach would involve full-scan LC-ESI-MS examination of deslorelin standards to determine whether the presumed oxidation and copper complexes can be separated. High-resolution mass spectrometry would be particularly useful in helping generate accurate mass molecular weights and molecular formulas. The point of these experiments is that alternative deslorelin structures are possible and most would be measurable by the clostripain/ProNHEt procedure unlike the LC-ESI-MS/MS method that relies on the intact unaltered peptide.

**Figure 12:**
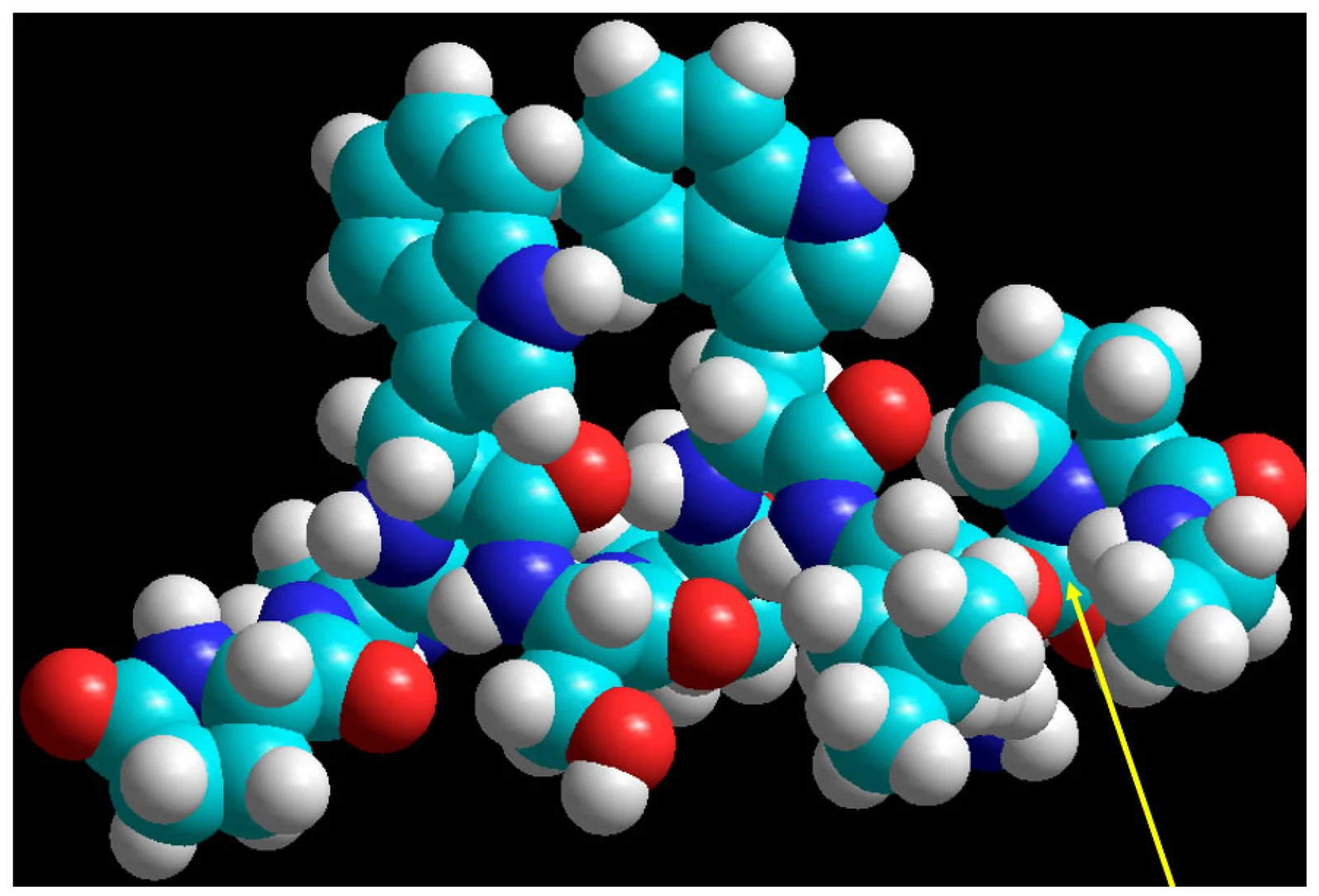
Deslorelin 3D structure optimized by Hyperchem molecular mechanics program with Amber force field, showing L-pyroglutamyl residue to left, and L-tryptophan and D-tryptophan residues (left and right) projecting out the top. Bond cleavage to release Pro-NHEt is indicated by the yellow arrow. Light blue = carbon atoms; dark blue = nitrogen atoms; red = oxygen atoms; white = hydrogen atoms.

Deslorelin was also studied by Hyperchem molecular mechanics modeling to give the three-dimensional representation shown in Figure 12 along with a designation of the clostripain cleavage site to enable visual orientation. The most striking feature of this model is the alignment of the Trp residues at the top of the image. This could potentially have implications for mechanisms capable of altering peptide function, as has been shown wherein tryptophan residues can react with one another to form new intermolecular bonds through dimerization [46]. This specific interaction has been documented in human superoxide dismutase 1, where tryptophan radicals generated by oxidative stress link monomers together, potentially altering protein function or stability [47].

## 4. Discussion

Deslorelin has many qualities that make it an attractive pharmaceutical option as a contraceptive, including a high safety record, low rate of negative side effects in most species, effectiveness, reversibility, and its potential for use in both males and females [48]. It is used primarily in veterinary medicine for reproductive management, including fertility control in dogs, treatment of adrenal disease in ferrets, and induction of ovulation in mares [4,49]. Despite its reputation for inherent safety, the hormonal properties of deslorelin acetate are suspected of being capable of reproductive toxicity and potential harm to unborn children or breast-fed children [50]. It is listed as a Category 1B reproductive toxin, meaning it may damage maternal fertility or the health of the unborn child with developmental toxicity pertaining to adverse toxic effects to the developing embryo or fetus [51]. As a GnRH agonist, overdose or improper handling of deslorelin can lead to significant hormonal imbalances, including initial surges in luteinizing hormone (LH) and follicle-stimulating hormone (FSH), followed by prolonged suppression of gonadal function [52]. In male animals, initial hormonal surges can lead to temporary increases in testosterone levels, which may cause behavioral changes or exacerbate conditions like benign prostatic hyperplasia before the hormone levels decline. In female animals, the initial surge in estradiol may cause signs of estrus or other hormone-related behaviors, while long-term use can lead to a prolonged suppression of reproductive function, which can impact fertility even after the treatment is discontinued [53]. Liman et al. [54] have recently reported that canine testicular expression of osteopontin was higher with deslorelin treatment than in untreated adult dogs. This is thought to have toxicological relevance in that osteopontin is a multifunctional, highly phosphorylated extracellular matrix protein that regulates bone remodeling, immune responses, and tissue repair. Under normal conditions, it is minimally expressed but accumulates rapidly during inflammation, injury, or malignancy to affect cell adhesion, migration, and survival [55,56]. In an early review [57], Pedula pointed out that GnRH analogs have beneficial effects in treatment of prostate cancer, but that an acute “flare” effect in plasma testosterone levels can occur due to the initial pituitary induction of LH and FSH release. This acute “flare” effect can result in a temporary worsening of existing signs with increased bone pain in up to 20% of patients. Pedula argued in favor of development of antagonists to alleviate the “flare” effects, but antagonists with D-Arg^6^ substitutions induced severe histamine release associated with edema of the face and extremities and some anaphylactoid reactions. Canine hormonal neutering with deslorelin combined with concurrent cyproterone injection induced regurgitation, diarrhea and decreased general condition in at least one case report [58]. Symptomatic treatment of GI difficulties followed by prednisolone therapy improved the situation, but lethargy persisted until removal of the deslorelin implant. Another study examined the usefulness of deslorelin for conditions other than induction of temporary infertility, such as benign prostatic hyperplasia and urinary incontinence [59]. Treatment duration lasted between 2 and 9 years and one-fourth of intact canine males had flare-up reactions. Two dogs developed neoplasia, and of these a bladder carcinoma was considered a result of deslorelin-induced gonadectomy. In another study [60] histological changes were studied in puppies treated with deslorelin for up to thirty weeks for postponement of canine puberty. Gonadectomy enabled histomorphometric examination, and two of six males presented with bilateral cryptorchidism. Study of testes revealed that tubular diameter and germinal epithelium height and composition were significantly decreased in deslorelin-treated males; similar study of ovarian structures revealed no differences in deslorelin-treated females. In summary, deslorelin is generally safe but subtle toxic effects are nonetheless evident. Therapeutic drug monitoring may be useful in assuring that deslorelin serum levels do not greatly exceed therapeutic levels such as those reported recently in treated goats, 83 +/− 28 ng/mL at C_max_ (maximum concentration post-treatment initiation) or 23.2 ng/mL at steady state [18].

Therapeutic drug monitoring is important in that it enables measurement of serum values of active pharmaceuticals to assure that their quantitative values do not reflect possibilities of toxicity or underdosage depending on the active range of therapeutic concentrations. However, the GnRH synthetic analogs have been engineered to avoid ordinary physiological effects that lead to metabolism or excretion, and with long half-lives such analogs have activity at very low levels. In such cases, it is important to have a means of determining the presence or absence of the compound either to assure ongoing therapeutic benefits or the drug’s absence in case of needed changes in an animal’s reproductive status. ELISA methods are possible approaches since they often have high sensitivity, but in general they suffer from low specificity and poor linearity. The approach proposed in this paper uses the low backgrounds and high specificities offered by tandem quadrupole mass spectrometers and their associated MRMs and has been thoroughly investigated for maximizing sensitivities from the aspects of handling clostripain, maximizing extraction efficiencies, introducing useful catalysts or accelerators, and optimizing GC and MS parameters wherever possible.

The milestones achieved during this research include the following: (1) owing to its Cu-chelating abilities, trientine was discovered to assist in the yield of ProNHEt from deslorelin; (2) HCl addition improved recovery of ProNHEt during concentration by nitrogen evaporation, specifically by creating a difficult-to-evaporate ProNHEt-HCl salt; (3) the antioxidant BHT was found to improve overall yield of ProNHEt; (4) addition of serum aided in the efficiency of the clostripain reaction; (5) the pH optimum for clostripain reactivity was between 7.2 and 7.8; and (6) the MTBSTFA reaction product, ProNHEt-tBuDMS, was the most specific and thermodynamically stable derivative. In addition, a GC-MS/MS method was optimized for detection of ProNHEt-tBuDMS.

A recent method [18] designed in our lab involves sensitive examination and quantitation of intact deslorelin in animal serum samples by Ultra-Performance Liquid Chromatography–Electrospray Ionization–Tandem Mass Spectrometry (UPLC-ESI-MS/MS). However, it should be kept in mind that one aspect of deslorelin chemistry is that it may exist in a variety of metabolic forms, each of which bears some level of the unaltered drug’s hormonal properties while still thwarting the excretory functions of the body. It would be difficult to measure these individual compounds for a total picture of GnRH activity, particularly with a lack of available standards. In this case it would be useful to combine the proposed method of ProNHEt determination with the UPLC-ESI-MS/MS method to provide quantitation of total intact deslorelin and—by subtraction—a characterization of total deslorelin breakdown products. This approach assumes lack of degradation-induced alteration of the Arg-ProNHEt portion of a given GnRH analog. One can see by the MALDI work reported here that oxidative reaction products seem to exist even in the standard used for deslorelin’s quantitation. Work has also not yet been carried out to examine standards and/or patient serum samples for the presence of copper-bound deslorelin as a complicating factor in analyses. Variations in potential MALDI-detected copper-bound deslorelin species in Figure 11 suggest that copper cations may be prone to dilution, a property which may well extend to deslorelin in the UPLC mobile phase, but this has not been definitively determined.

Certain factors can accelerate the oxidation of pharmaceutical peptide hormones, including the presence of reactive oxygen species, the presence of catalytic trace metal ions such as iron and copper, photooxidation by exposure to UV and visible light that particularly affect aromatic residues (TRP, TYR, HIS), elevated temperatures and prolonged exposure to atmospheric oxygen [61]. The amino acid residues most susceptible to oxidation in pharmaceutical peptide hormones are methionine, cysteine, histidine, tryptophan, and tyrosine; phenylalanine is also listed as oxidation-prone though generally less reactive than the other aromatic residues mentioned [62]. In deslorelin, peptide oxidation is possible at TRP, TYR and HIS residues. Tryptophan is highly susceptible to oxidation by reactive oxygen species (ROS), including hydroxyl radicals, singlet oxygen, and ozone, which react at diffusion-limited or significant physiological rates in vivo. The primary chemical mechanism involves the abstraction of a hydrogen atom from the indole ring to form a tryptophanyl radical, which subsequently reacts with oxygen or superoxide to generate unstable hydroperoxides [63]. At least three oxidized species could be observed in deslorelin by MALDI and the compound has four residues that can oxidize; however, a simpler explanation may be that the *m*/*z* 1300 species in Table 3 is the result of a single hydroxylation event, the *m*/*z* 1316 species is a peroxide or sulfone, and the *m*/*z* 1332 species is a combination of the two. Remember that MALDI does not utilize chromatography, such that each of these species may comprise more than one structure, although focus may be on the TRP residues as the most reactive. The MALDI findings of Figure 10 show that ProNHEt can be released from all versions including degraded or oxidized forms of deslorelin. This is evident in that all species were significantly reduced or gone following clostripain treatment, such that the oxidized and/or copper-bound versions of the drug did not accrue any unanticipated immunity to the effects of the enzyme.

Note that adjacent TRP residues as in deslorelin (Figure 12) have also been described as capable of reacting with one another. The interactions can take the forms of (1) electron transfer, and (2) dimerization following radical formation [64]. The electron transfer or tunneling takes the form of “hole hopping” which enables electrons to move efficiently between distant redox centers, whereas radical formation as from reactive oxygen species can lead to dimerization, a process that may protect proteins from further oxidative damage. New intermolecular bonds in the latter mechanism can reduce the molecular weight of a structure such as deslorelin by two, but MALDI may not have the resolving power to enable discernment of a *m*/*z* 1281 species from the principal 1283 species.

The described ProNHEt method has the advantage that it can quantitatively determine deslorelin forms that include intact carboxy termini in a 1:1 molar ratio to the parent compound, assuming that enzymatic reaction conditions are appropriately optimized. MALDI examination of deslorelin before and after clostripain digestion as shown in Figure 10 revealed traces of the undigested deslorelin species; however, comparison of intensities before and after digestion suggests a residual amount on the order of 5%, indicating a roughly 95% enzymatic efficiency which should suffice for the intended total quantitation. Note that this represents a rough approximation, since two different molecular species are involved and the MALDI method does not necessarily provide a good means of relative compound quantitation.

**Figure 13:**
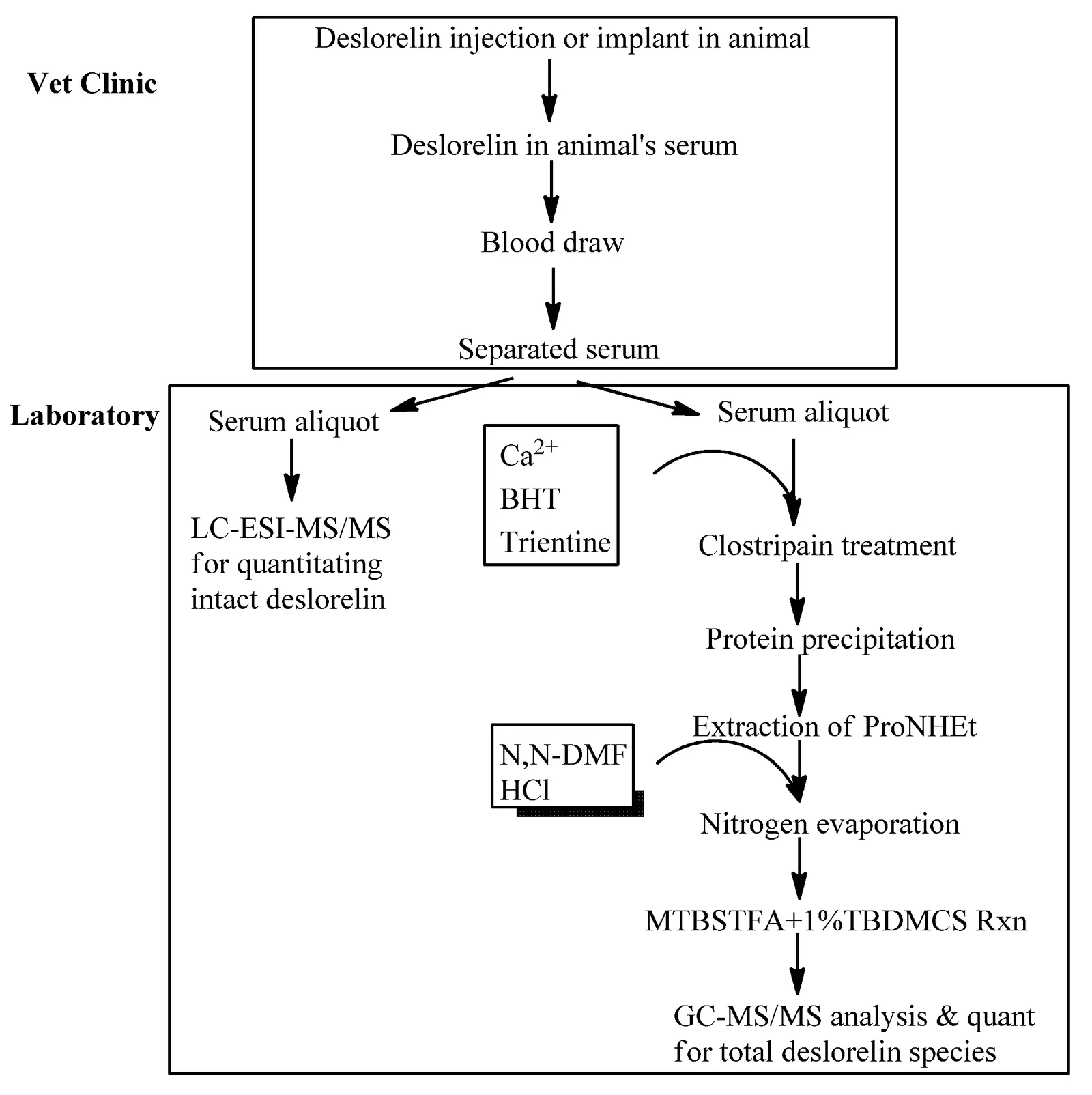
Scheme for the entire process of quantitating deslorelin (or alternative ProNHEt-bearing GnRH analog) by clostripain digestion of serum from an animal treated in a vet clinic or in the field.

The overall method should be extendible to other GnRH compounds in Figure 1 that include the ProNHEt structure at the carboxy terminus, including buserelin, histrelin, leuprorelin, and fertirelin. Figure 13 summarizes the overall process in schematic form.

Examination was not made of possible species-specific differences in serum composition and how that might affect performance of the described assay. Bovine serum was the sole target matrix. Differences have been found in serum that could potentially have significant effects. For example, some authors have explicitly considered species-specific differences in enzymatic digestion and metabolism of serum proteins. Götz [65] demonstrated that each animal species possesses characteristic protein distribution patterns in its serum, with species-bound peculiarities affecting specific enzymatic activities such as esterases, amylases, and alkaline phosphatases. Kuzmina et al. [66] carried out comparative physiology studies that highlight significant interspecies variations in digestive enzyme dynamics, such as trypsin activity, which varies markedly between poultry, mammals, and rodents. Thus, the clostripain approach described here would need validation in any animal species of interest.

Treatment of serum containing deslorelin as shown in Figure 9 allowed us to determine that deslorelin was the sole source of ProNHEt in examined samples. In other words, serum did not generate ProNHEt nor did clostripain self-digestion, if possible, nor did deslorelin in the absence of enzymatic treatment. However, logistics of funding or manpower did not enable us to carry out the next steps which would involve (1) examination by LCMSMS of intact deslorelin and its correspondence stoichiometrically with ProNHEt measured following enzymatic cleavage, and (2) measurements in samples from treated zoo animals or other relevant specimens. The hope is that other laboratories interested in these measurements will come forward and apply the described method, possibly in collaboration with the MSU VDL Toxicology lab which has the UPLC-ESI-MS/MS method for intact deslorelin in place. Note that an uninvestigated approach may simply be direct UPLC-ESI-MS/MS measurement of ProNHEt, bypassing the needs for derivatization and GC-MS/MS operation.

It should be stressed that measurement of released ProNHEt alone cannot unequivocally identify deslorelin when another ProNHEt-containing analog could be present. In most cases it would be expected that the patient history will exactly describe the GnRH analog being applied. However, in dairy and beef cattle, for example, a standard protocol often involves two GnRH injections. The first induces a new follicular wave, and the second, given after prostaglandin administration, synchronizes ovulation for timed artificial insemination [67]. Since the various GnRH analogs have different potencies depending on molecular structure, there may be occasions to give combinations of buserelin and gonadorelin to cows for strategic induction of LH surges [68]. For reference, different GnRH analog potencies are roughly as follows: (1) Histrelin: 150–200 times more potent than native GnRH; (2) Deslorelin: ~100 times that of native GnRH; (3) Triptorelin: 100-fold more potent than native GnRH and six times more potent than Buserelin; (4) Buserelin: ~20 times more potent than native GnRH; (5) Leuprorelin: equipotent to Triptorelin; (6) Fertirelin: potency is comparable to other standard GnRH agonists like Leuprorelin [69,70,71,72].

## 5. Conclusions 

In conclusion, the proposed method should provide useful tools for quantitating GnRH analogs that occur at low concentrations in treated animal serum. It would be of value to determine the extent to which determination by the ProNHEt surrogate corresponds to separate LC-MS/MS quantitation of intact deslorelin or other GnRH analogs that terminate in the N-ethyl-modified proline (ProNHEt). Much of the validation work presented here focused on GC applicability; much of this would become of less interest if the released ProNHEt was measured directly by LC-MS/MS. Such an approach would render the low recovery from liquid–liquid extraction a nonissue, since serum could be simply protein precipitated and the resultant supernatant measured by direct injection. It was useful to see how well the clostripain digestion was able to handle relatively large concentrations of deslorelin such as in the experiments illustrated in Figure 9. Nevertheless, study needs to be carried out in clinically incurred samples and low-level spikes to verify applicability to real-life samples.

## Figures and Tables

**Table 1:** MRM settings used for ProNHEt-tBuDMS detection on the GC-MS/MS.

Quantitation MRM (*m*/*z*)	Collision Energy (eV)
184.1 → 73.0	20
Qualifier MRM (*m*/*z*)	Collision Energy (eV)
199.1 → 75.0	21
199.1 → 102.1	23
199.1 → 125.1	15
199.1 → 130.1	12
199.1 → 181.1	11
241.2 → 75.0	16
241.2 → 102.1	15
241.2 → 125.1	16
241.2 → 172.1	16
241.2 → 184.1	15

**Table 2:** Expectations regarding analytical presence or absence of ProNHEt following incubation of the listed mixtures prior to analysis with results shown in Figure 9.

Mixture	Expect ProNHEt	Did We Get ProNHEt?
Clostripain and deslorelin	YES	YES
Clostripain alone (self-digestion)	no	no
Clostripain and serum	no	no
Deslorelin alone	no	no
Serum alone	no	no
Clostripain and ProNHEt	YES	YES

**Table 3:** Interpretations of deslorelin mass spectra acquired by MALDI. To the left are measured *m*/*z* values and their interpretations. Expected cleavage product *m*/*z* values are shown in the third column and whether the corresponding cleavage product was observed in MALDI.

m/z	Interpretation	m/z Minus ProNHEt Plus OH	Cleavage Product Found?
*m*/*z* 1284	Deslorelin + H	1159	yes
*m*/*z* 1300	Deslorelin + O	1175	yes
*m*/*z* 1305	Deslorelin + Na	1180	yes
*m*/*z* 1316	Deslorelin + O_2_	1191	yes
*m*/*z* 1332	Deslorelin + O_3_	1207	yes
*m*/*z* 1338	Deslorelin + O_2_ + Na	1213	yes
*m*/*z* 1347	Deslorelin + Cu	1222	yes
*m*/*z* 1370	Deslorelin + Cu + Na	1245	yes
*m*/*z* 1384	Deslorelin + Cu + K	1259	no

## Data Availability

The data presented in this study are available on request from the corresponding author.

## Supplementary Materials

The following supporting information can be downloaded at: https://www.mdpi.com/article/10.3390/toxics14100913/s1, Figure S1: Collision energy optimization for the proposed ProNHEt-tBuDMS MRMs. Examination of MRMs occurred by 5 eV increments.; Figure S2. Ratios of qualifier MRMs to the quantifier MRM *m/z* 184→73 for ProNHEt-tBuDMS. Figure S3. Temperature for optimal MTBSTFA reaction: 60 °C [left] vs 70 °C [right]. Figure S4. GC-MS/MS parameters optimized for maximum sensitivity for ProNHEt-tBuDMS detection. A, intensity versus nitrogen flow (mL/min) to the collision cell for collision-induced dissociation; B, effect of increasing gain in the 10–40 range for optimal electron multiplier response; C, variation of GC inlet temperature across the 160–230 °C. range for optimal response; D, variation of the initial GC oven temperature across the 60–150 °C range for optimal response. These studies were carried out a single time. Figure S5. Standard curve demonstration with derivatized neat ProNHEt standard across the 10–10,000 ppb concentration range to show linearity of response at a MS gain setting of 30. Figure S6. ProNHEt MRM area ratios summary. Figure S7. Quantifier MRM *m/z* 184.1 → 73.0 for ProNHEt standards at 1.0 (left) and 0.1 (right) ppb. Figure S8. Plot of residuals (as nominal minus refit value) for three ProNHEt standard curves (A, B, C) versus nominal concentration in ppb. Figure S9. ProNHEt-tBuDMS responses with and without added trientine during the cleavage reaction. Figure S10. Effects of addition of 0.05% BHT as an antioxidant to the standard reaction mix under various conditions. Figure S11. Quantitative representation of the area counts of each reaction product at different amounts of serum to the standard reaction mix. Figure S12. Temperature dependence of the clostripain enzymatic digestion. Shown are ProNHEt-tBuDMS *m/z* 184→73 area counts versus temperature, °C. Figure S13. Effects of addition of the labeled # µL 0.1 M HCl to ~8 mL acetonitrile extract from ProNHEt reaction mix during the nitrogen evaporation step, indicating optimal recovery on addition of 150 µL. Figure S14. Corrections to measured MALDI values. Top, peptides used to calibrate MALDI, and calibration curve for recalculating unknowns. Figure S15. Initial MALDI study with increasing concentration 1, 10, 100, 1000 ppm, left-to-right. Figure S16. Linear responses of deslorelin intensity as *m/z* 1284 and its principal sodium adduct as *m/z* 1306 as determined by MALDI. Table S1. Interday precision for determination of ProNHEt. Average of eight studies. Table S2. Intraday precision for determination of ProNHEt. Average of three studies. Table S3. Specificity determination for ProNHEt qualifier MRMs. Table S4. Recovery experiment for ProNHEt. Standards extracted from serum were backfit into an unextracted standard curve in the range 500–10,000 ng/mL. Table S5. Recipes for reaction mixtures intended to evaluate the effectiveness of the clostripain approach to deslorelin measurement. Table S6. Adjustments to MALDI-derived *m/z* values. A, list of MALDI standards including their molecular weights, monoisotopic masses, the expected *m/z* values in the presence of formic acid, the values measured by MALDI and the resulting errors in amu (atomic mass units) (measured minus expected values). B, application of the adjusted *m/z* values as an adjustment correlation to two measurements of deslorelin; a measured value of *m/z* 1284.400 is converted to 1283.925 resulting in an error of 1.2849 amu from the expected value shown at the bottom.

## Abbreviations

The abbreviations used are: BSA, bovine serum albumin; Clostripain, Clostridiopeptidase B; Da, Dalton; DMF, N,N-dimethylformamide; DTT, 1,4-dithiothreitol; ELISA, enzyme-linked immunosorbent assay; GC-MS/MS, gas chromatography–tandem quadrupole mass spectrometry; GnRH, gonadotropin-releasing hormone; HPLC, high-performance liquid chromatography; MALDI-TOF, matrix-assisted laser desorption/ionization–time-of-flight; MRM, Multiple Reaction Monitoring; MTBSTFA, N-(tert-butyldimethylsilyl)-N-methyltrifluoroacetamide); TBDMSCl, tert-butyldimethylsilyl chloride; ppb, parts per billion; ProNHEt, modified proline moiety released by enzymatic cleavage (N-ethyl-2-pyrrolidinecarboxamide).
