# Supplementary material for: Proposed Method for Mass Spectrometric Measurement of the Gonadotropin-Releasing Hormone Agonist Deslorelin
Source: Toxics. 2026 Oct 16;14(10):913. doi: 10.3390/toxics14100913 (PMC13709457; doi:10.3390/toxics14100913)
Supplement: Supplementary file 1 [file toxics-14-00913-s001.zip › toxics-4566705-supplementary.pdf]

Proposed Method for Mass Spectrometric Measurement of the Gonadotropin-Releasing Hormone Agonist Deslorelin

Christine Wei [1], John P. Buchweitz [2,3], Rinosh Mani [4], Dalen Agnew [2] and Andreas F. Lehner [3,*]

[1] Department of Comparative Medicine and Integrative Biology, College of Veterinary Medicine, Michigan State University, East Lansing, MI 48824, USA; weizimu@msu.edu
[2] Pathobiology & Diagnostic Investigation, College of Veterinary Medicine, Michigan State University, East Lansing, MI 48824, USA; buchwei2@msu.edu (J.P.B.); agnewd@msu.edu (D.A.)
[3] Michigan State University Veterinary Diagnostic Laboratory, Section of Toxicology, College of Veterinary Medicine, Michigan State University, 4125 Beaumont Rd, East Lansing, MI 48910, USA
[4] Michigan State University Veterinary Diagnostic Laboratory, Section of Bacteriology, Michigan State University, East Lansing, MI 48910, USA; manirino@msu.edu
* Correspondence: lehnera@msu.edu

Figures

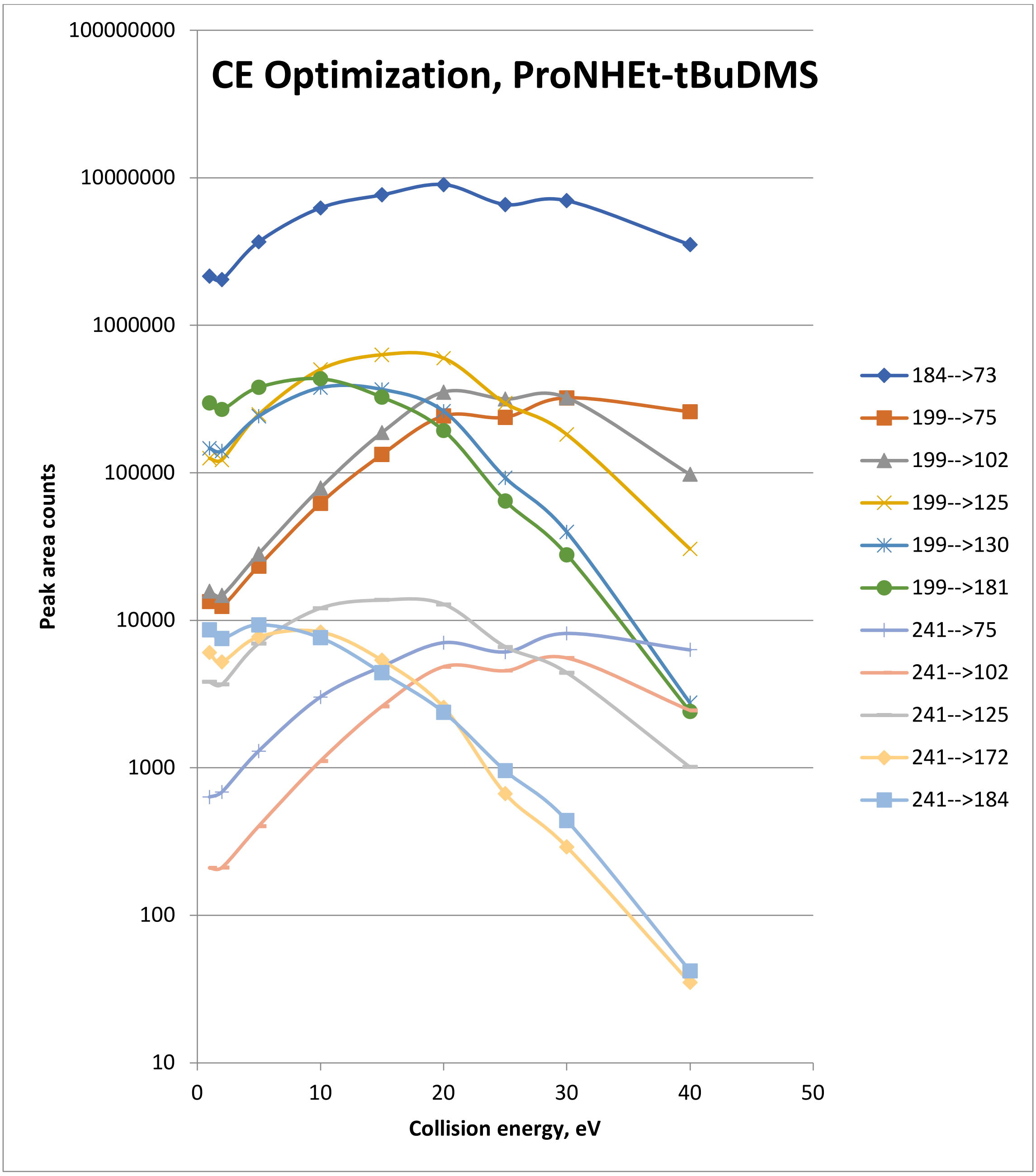

Figure S1. Collision energy optimization for the proposed ProNHEt-tBuDMS MRMs. Examination of MRMs occurred by 5 eV increments. Reexamination by 1 eV increments did not alter the findings of optimum CE depicted here. Note that the y-axis is shown in logarithmic format to enable easier visualization of low abundance MRMs. This study was the result of a single experiment.

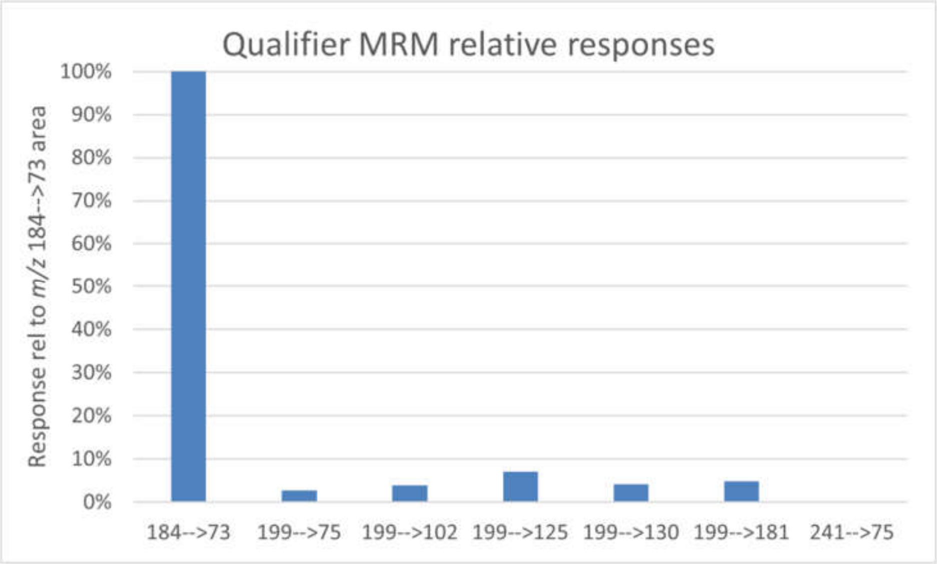

Figure S2. Ratios of qualifier MRMs to the quantifier MRM *m/z* 184→73 for ProNHEt-tBuDMS. This was the result of a single experiment.

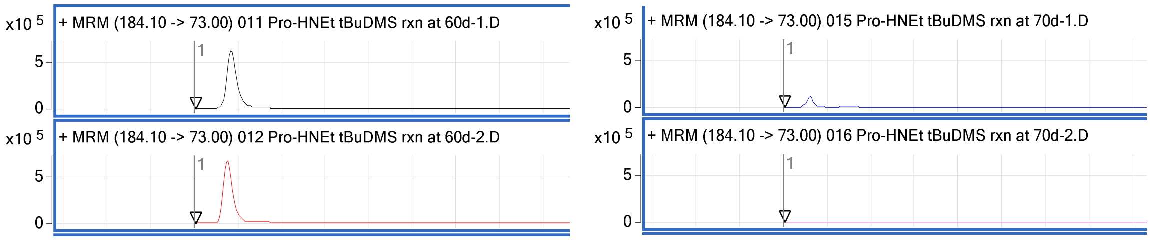

Figure S3. Temperature for optimal MTBSTFA reaction: 60 °C [left] vs 70 °C [right]. Each derivatization was done in duplicate, with chromatographic traces for *m/z* 184 → 73 shown.

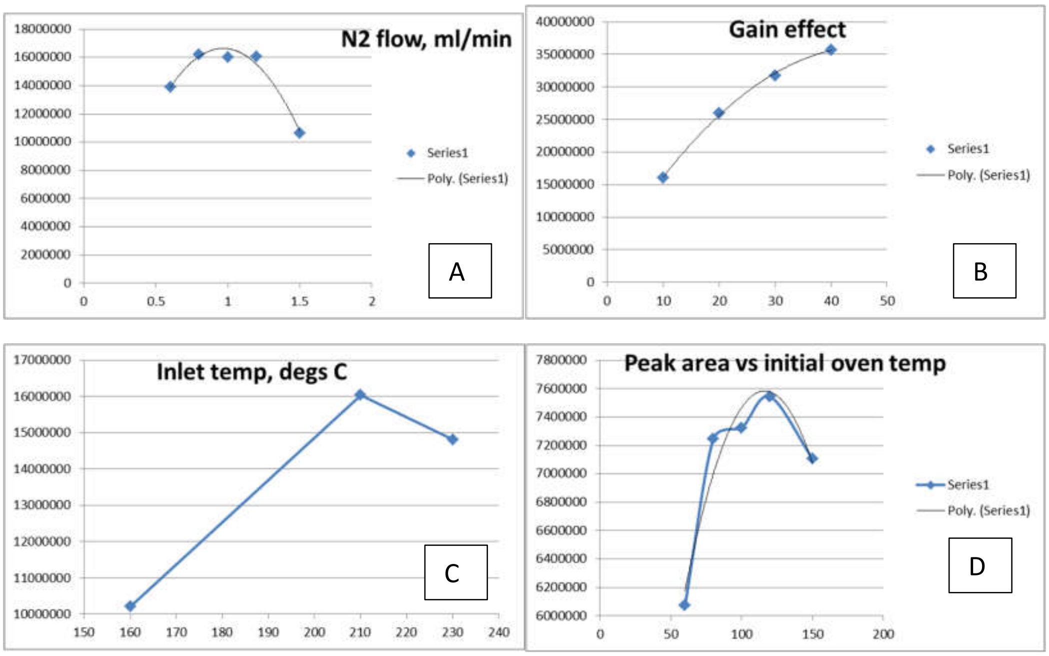

Figure S4. GC-MS/MS parameters optimized for maximum sensitivity for ProNHEt-tBuDMS detection. A, intensity versus nitrogen flow (mL/min) to the collision cell for collision-induced dissociation; B, effect of increasing gain in the 10 – 40 range for optimal electron multiplier response; C, variation of GC inlet temperature across the 160 – 230 ^{o}C. range for optimal response; D, variation of the initial GC oven temperature across the 60 – 150 ^{o}C range for optimal response. These studies were carried out a single time.

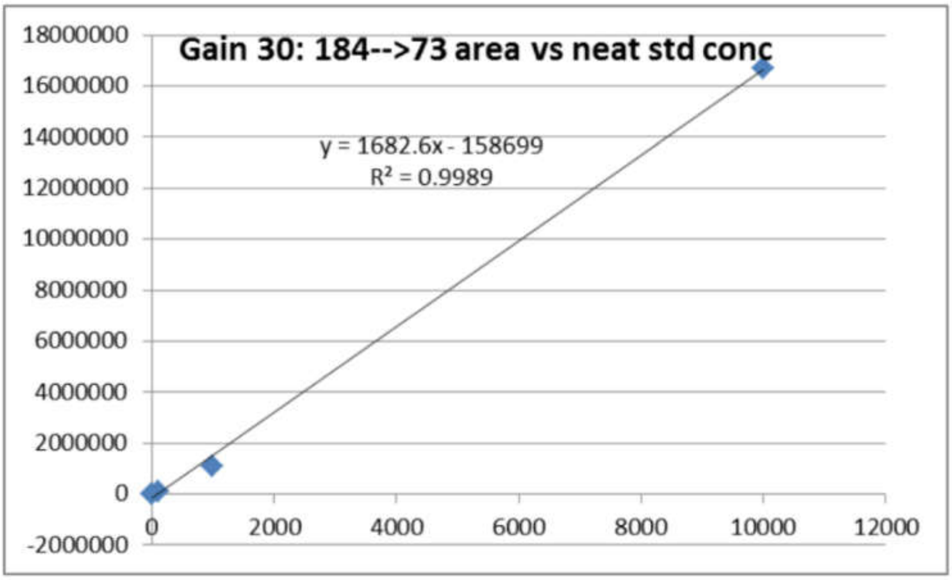

Figure S5. Standard curve demonstration with derivatized neat ProNHEt standard across the 10 – 10000 ppb concentration range to show linearity of response at a MS gain setting of 30. The standard curve was established by plotting the total peak area values for the *m/z* 184→73 MRM of individual standards on the y-axis versus their respective ProNHEt concentrations on the x-axis. This study resulted from a single experiment.

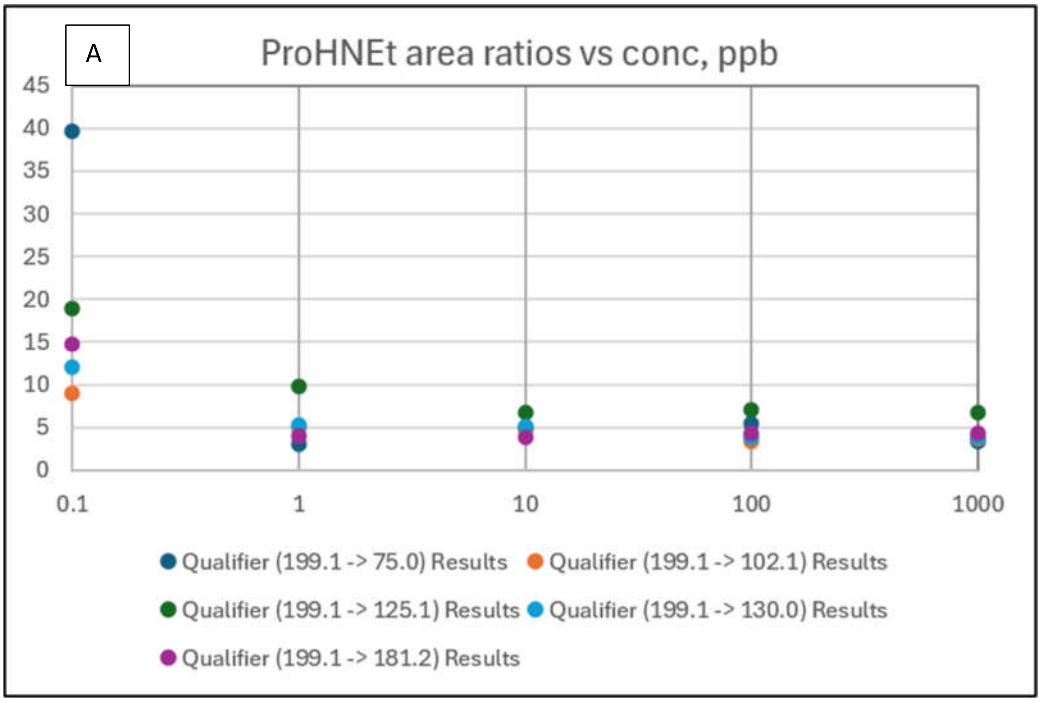

B

Pro-HNEt tBuDMS, ppb	Qualifier (199.1 -> 75.0) Results	Qualifier (199.1 -> 102.1) Results	Qualifier (199.1 -> 125.1) Results	Qualifier (199.1 -> 130.0) Results	Qualifier (199.1 -> 181.2) Results
0.1	39.6	9.0	18.9	12.0	14.8
1	3.1	4.9	9.8	5.3	4.0
10	4.9	4.6	6.7	5.2	3.9
100	5.5	3.4	7.1	3.9	4.3
1000	3.4	3.6	6.7	3.9	4.3

Figure S6. ProNHEt MRM area ratios summary. A, ProNHEt measured area ratios for qualifier ions versus concentration (ppb). Note logarithmic plot of the x-axis. B, tabulation of the individual area ratios for each concentration differing by 10-fold in the 0.1 – 100 ppb range. These findings resulted from a single study.

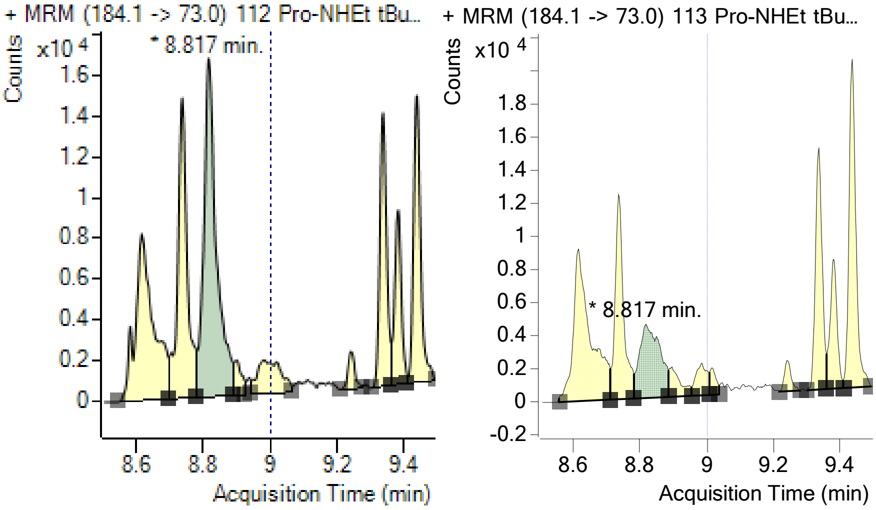

Figure S7. Quantifier MRM *m/z* 184.1 → 73.0 for ProNHEt standards at 1.0 (left) and 0.1 (right) ppb. The target peaks are shaded in green. Manually determined S/N ratios by area counts were 54.7 and 33.7, respectively.

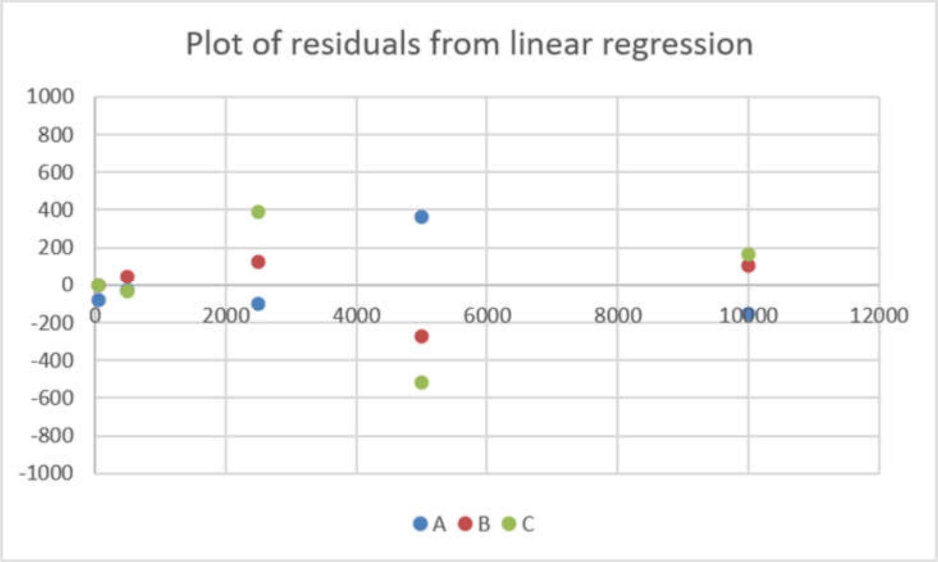

Figure S8. Plot of residuals (as nominal minus refit value) for three ProNHEt standard curves (A, B, C) versus nominal concentration in ppb.

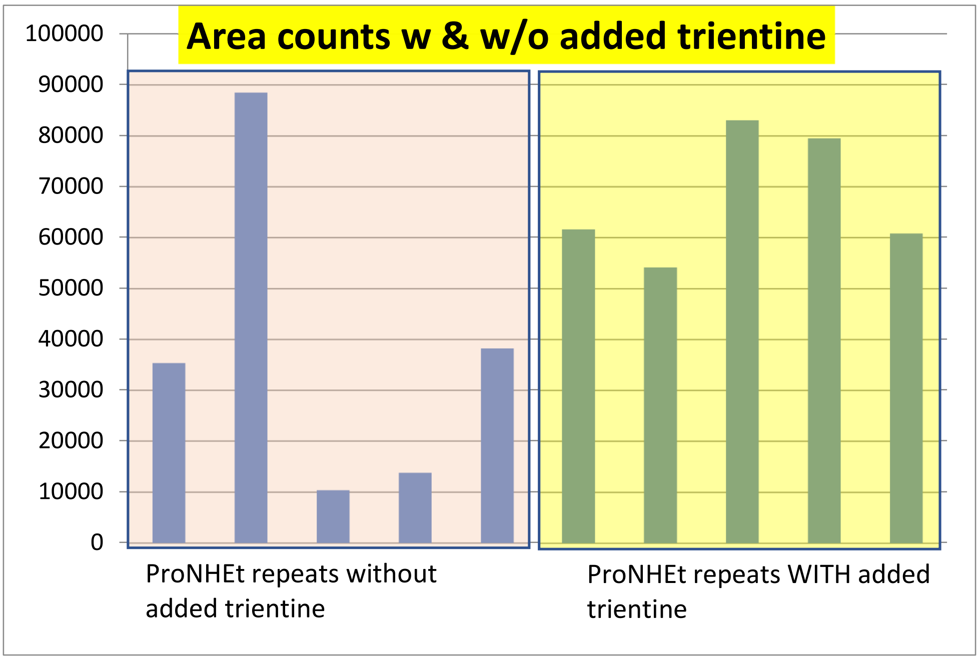

Figure S9. ProNHEt-tBuDMS responses with and without added trientine during the cleavage reaction. The left group shows five independent extractions without addition of trientine in the sample, whereas the right group shows five independent extractions with trientine added. Average recovery with deslorelin alone: 30.1% +/- 12.1%; average recovery with trientine added: 42.5% +/- 3.3%.

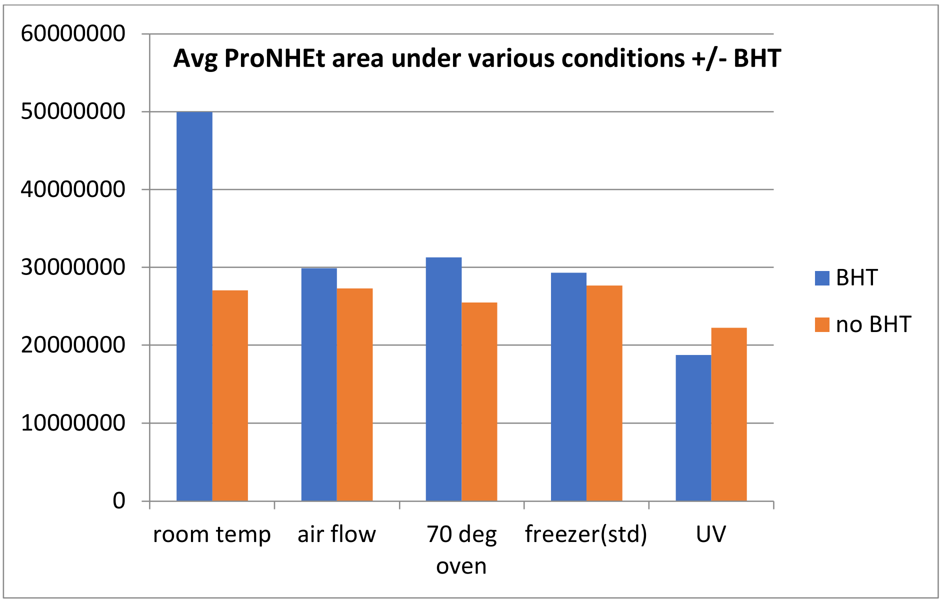

Figure S10. Effects of addition of 0.05% BHT as an antioxidant to the standard reaction mix under various conditions. Shown are the measured ProNHEt-tBuDMS chromatographic area counts for each condition, including simple room temperature (25 °C) incubation of the Clostripain reaction, the same incubation with air flow across an open reaction tube, the same reaction followed by 0.5 hr storage in a 70 °C oven, inclusion of a freezer step in the standard ProNHEt extraction method, or shaking the clear reaction tube in the presence of a UV light source. This was the result of a single study.

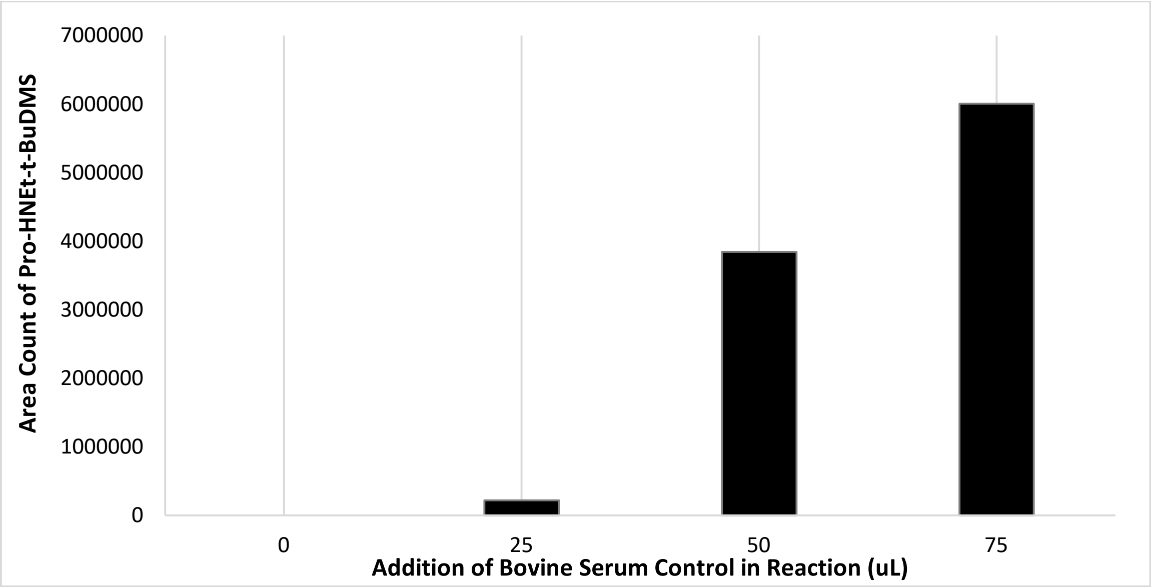

Figure S11. Quantitative representation of the area counts of each reaction product at different amounts of serum to the standard reaction mix. This study resulted from a single experiment.

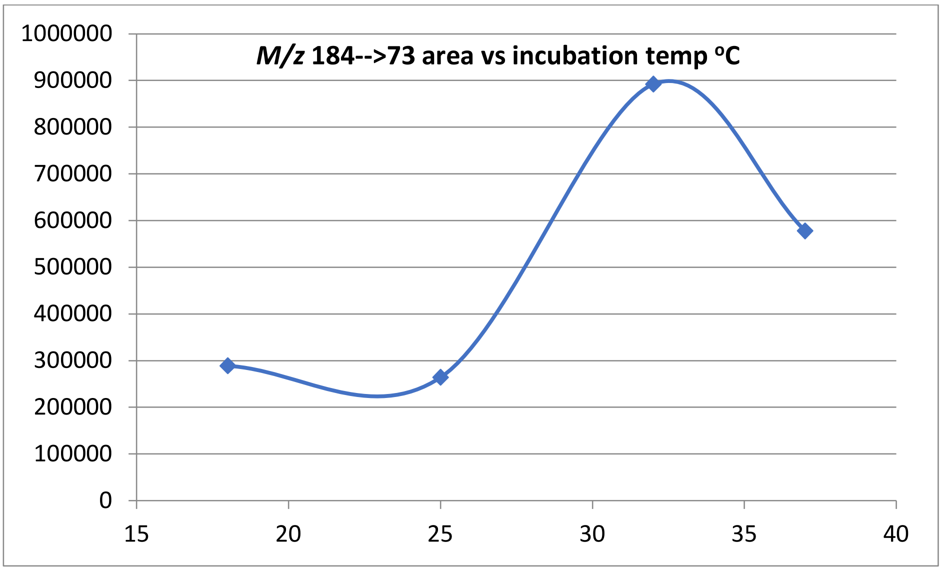

Figure S12. Temperature dependence of the clostripain enzymatic digestion. Shown are ProNHEt-tBuDMS *m/z* 184→73 area counts versus temperature, °C. This finding was the result of a single study.

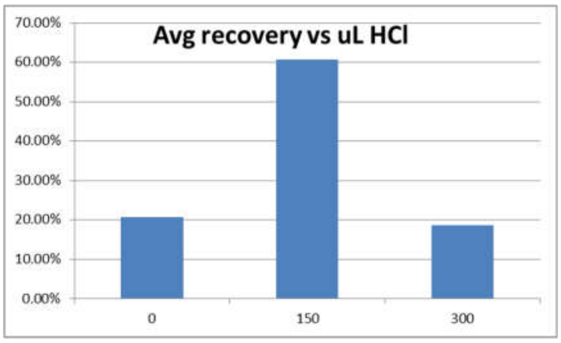

Figure S13. Effects of addition of the labeled # µL 0.1 M HCl to ~8 mL acetonitrile extract from ProNHEt reaction mix during the nitrogen evaporation step, indicating optimal recovery on addition of 150 µL. This study resulted from a single experiment.

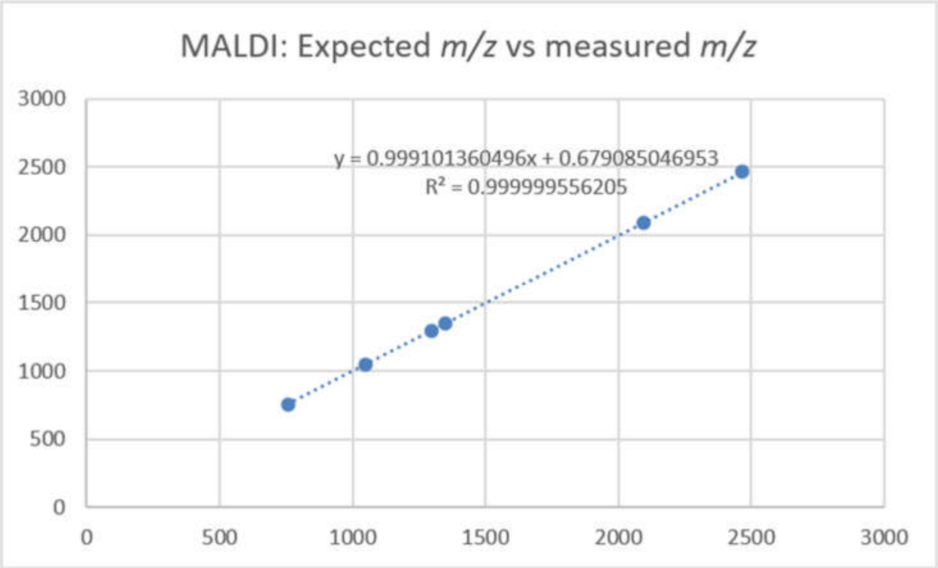

Composition: C64 H83 N17 O12

Nominal Mass: 1281

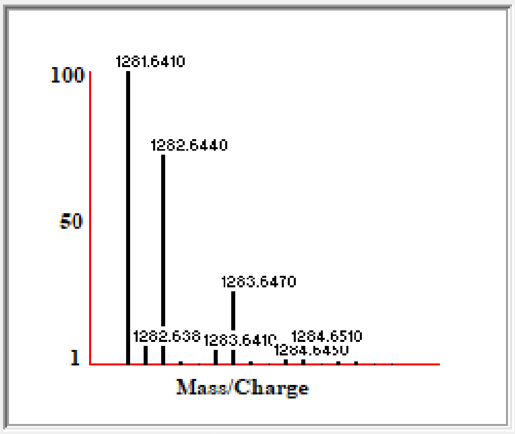

Mass	Rel Abund
1281.6406	100.00
1282.6377	6.31
1282.6440	71.18
1282.6450	0.48
1283.6348	0.19
1283.6411	4.49
1283.6475	24.94
1283.6484	0.34

Figure S14. Corrections to measured MALDI values. Top, peptides used to calibrate MALDI, and calibration curve for recalculating unknowns. Bottom, the accurate mass determination for the formula $C_{64}H_{83}N_{17}O_{12}$ for deslorelin by Mass Hunter Pro, showing an unprotonated monoisotopic mass of 1281.6406.

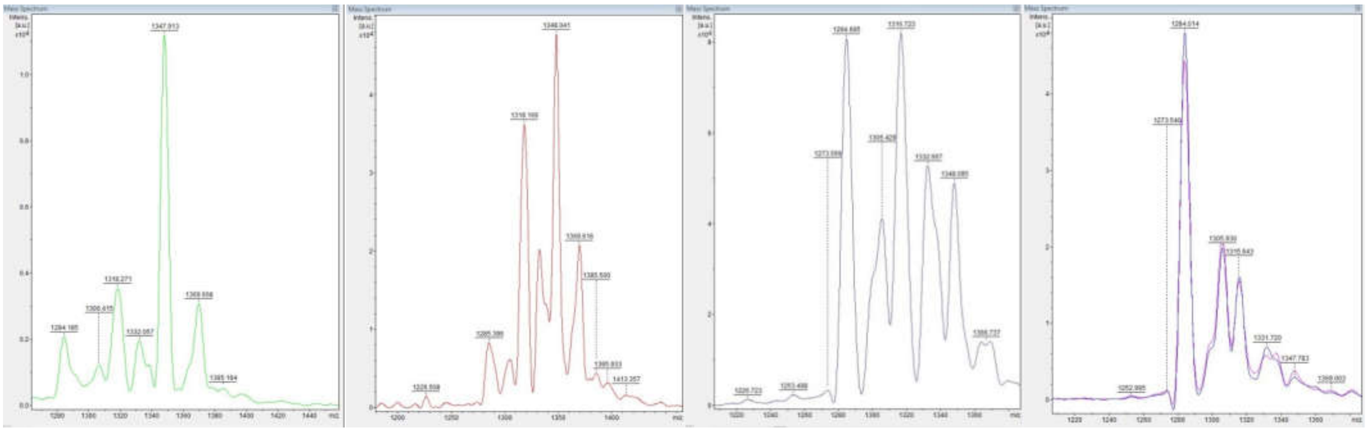

Figure S15. Initial MALDI study with increasing concentration 1, 10, 100, 1000 ppm, left-to-right.

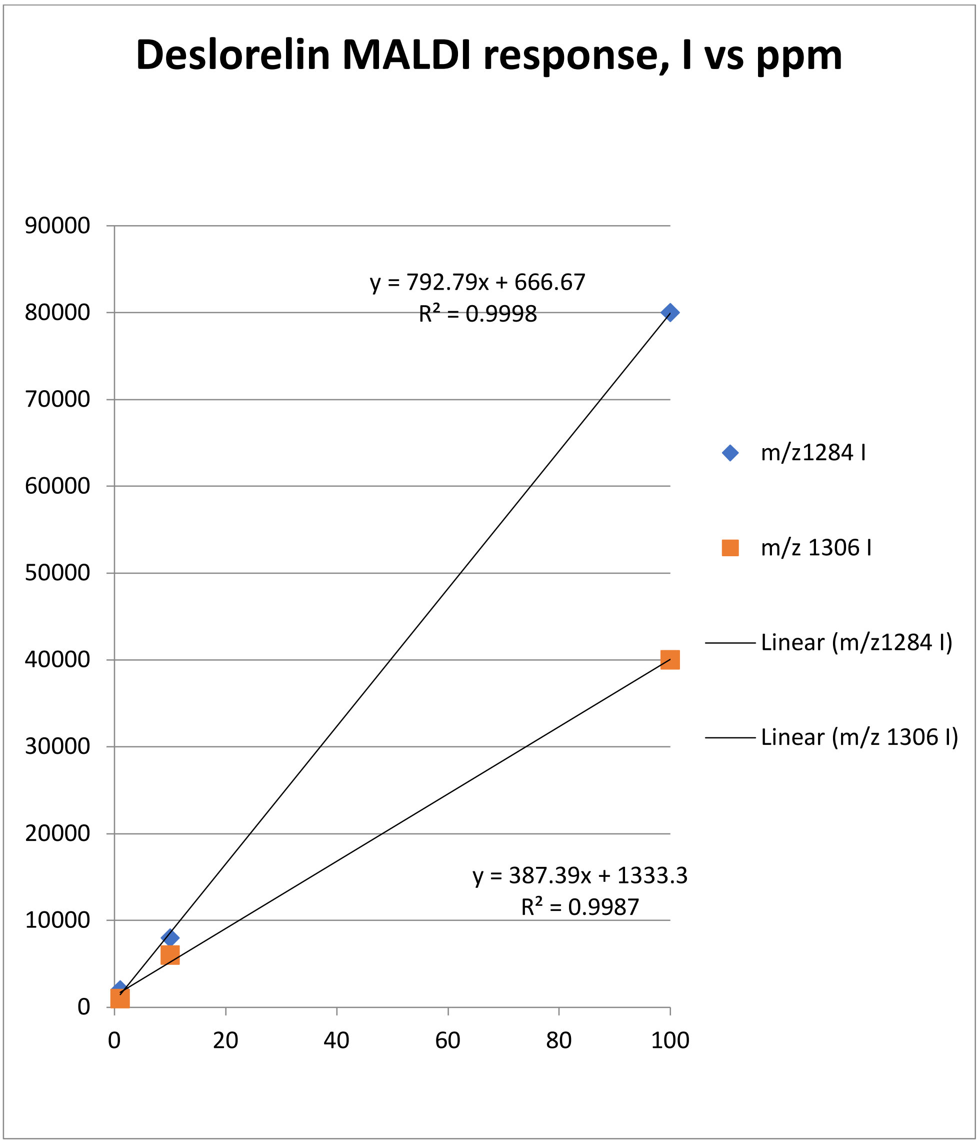

Figure S16. Linear responses of deslorelin intensity as *m/z* 1284 and its principal sodium adduct as *m/z* 1306 as determined by MALDI. Shown are intensity (I) vs concentration in ppm. This resulted from a single study.

Tables

Table S1. Interday precision for determination of ProNHEt. Average of eight studies.

ppb	Avg	Std dev	%RSD
10000	9942.1	99.4	1.00%
5000	5101.8	364.3	7.14%
2500	2542.9	224.8	8.84%
500	475.8	30.5	6.42%
50	51.7	2.3	4.50%

Table S2. Intraday precision for determination of ProNHEt. Average of three studies.

ppb	Avg	Std dev	%RSD
10000	10048.1	28.5	0.28%
5000	4746.1	185.3	3.90%
2500	2743.5	82.6	3.01%
500	460.6	30.4	6.61%
50	51.7	2.3	4.50%

Table S3. Specificity determination for ProNHEt qualifier MRMs. Listed are average percent values of the quantifier MRM, the standard deviation and resulting %RSD. Twenty-four determinations from a single day were included.

	Qualifier (199.1 -> 75.0) Results	Qualifier (199.1 -> 102.1) Results	Qualifier (199.1 -> 130.0) Results	Qualifier (199.1 -> 181.2) Results
Avg	49.22	53.09	56.65	61.33
Std dev	3.91	4.03	3.31	1.17
RSD	7.9%	7.6%	5.9%	1.9%

Table S4. Recovery experiment for ProNHEt. Standards extracted from serum were backfit into an unextracted standard curve in the range 500 – 10000 ng/mL. This enabled determination of recovery as the division of backfit values for extracted by the backfit values for the standards. The average value of 35% represents the range 2500 – 10000 ppb, assuming the 500 ppb result to be an outlier. This was the result of a single study.

ng/mL	Unextracted	Extracted	Extr/Unextr recovery
500	449.0	624.4	139%
2500	2816.2	1135.3	40%
5000	4711.1	1491.3	32%
10000	10052.4	3216.8	32%
		Average	35%

Table S5. Recipes for reaction mixtures intended to evaluate the effectiveness of the Clostripain approach to deslorelin measurement. All mixtures were incubated at 25 °C overnight.

Mixture*	Deslorelin	Serum	ProNHEt	Clostripain	Water	Buffer	DTT	$CaCl_2$	Total vol
Clostripain and deslorelin	100			500	150	50	100	100	1000
Clostripain alone (self-digestion)				500	250	50	100	100	1000
Clostripain and serum		100		500	150	50	100	100	1000
Deslorelin alone	100				650	50	100	100	1000
Serum alone		100			650	50	100	100	1000
Clostripain and ProNHEt			100	500	150	50	100	100	1000

*Components included 1 mg/mL deslorelin in water, bovine serum, 1 mg/mL ProNHEt in acetonitrile, 0.1 mg/mL Clostripain in storage buffer, 25 mM DTT in water, 10 mM CaCl2 in water and 200 mM Na2HPO4 as buffer, pH 7.4.

Table S6. Adjustments to MALDI-derived *m/z* values. A, list of MALDI standards including their molecular weights, monoisotopic masses, the expected *m/z* values in the presence of formic acid, the values measured by MALDI and the resulting errors in amu (atomic mass units) (measured minus expected values). B, application of the adjusted *m/z* values as an adjustment correlation to two measurements of deslorelin; a measured value of *m/z* 1284.400 is converted to 1283.925 resulting in an error of 1.2849 amu from the expected value shown at the bottom. On another occasion an adjusted value of *m/z* 1282.885 is closer to the expected value but still shows an error of 0.2449 from the expected value.

A.

**Standard**	**m.w.**	**Mono-isotopic masses**	**Mono-isotopic mass +H, *m/z* (expected)**	**Measured, *m/z***	**Error, measured minus expected**
Bradykinin 1-7	756.86	756.400	757.3997	757.487	0.0873
Angiotensin II	1046.18	1045.535	1046.5345	1047.3	0.7655
Angiotensin I	1296.50	1296.680	1297.68	1297.63	-0.05
Substance P	1347.63	1347.712	1348.7122	1349.18	0.4678
ACTH clip 1-17	2093.50	2093.086	2094.0862	2094.86	0.7738
ACTH clip 18-39	2465.70	2464.199	2465.1989	2467.18	1.9811

B.

Measured, *m/z*		Corrected, *m/z*	Error, amu
1284.400	convert to-->	1283.925	1.2849
1283.359	convert to-->	1282.885	0.2449
Expect	M+H	1282.64	
